# PCV2 Uptake by Porcine Monocytes Is Strain-Dependent and Is Associated with Amino Acid Characteristics on the Capsid Surface

**DOI:** 10.1128/spectrum.03805-22

**Published:** 2023-01-31

**Authors:** Yueling Ouyang, Hans J. Nauwynck

**Affiliations:** a Laboratory of Virology, Department of Translational Physiology, Infectiology and Public Health, Faculty of Veterinary Medicine, Ghent University, Ghent, Belgium; University of Prince Edward Island

**Keywords:** PCV2, PCVAD, PMWS, PDNS, abortion, peripheral blood monocyte, viral capsid, uptake, glycosaminoglycans, receptor, phosphacan

## Abstract

Porcine circovirus type 2 (PCV2) is associated with several economically important diseases that are described as PCV2-associated diseases (PCVADs). PCV2 is replicating in lymphoblasts, and PCV2 particles are taken up by monocytes without effective replication or complete degradation. Glycosaminoglycans (GAGs) have been demonstrated to be important receptors for PCV2 binding and entry in T-lymphocytes and continuous cell lines. The objective of this study was to determine whether differences exist in viral uptake and outcome among six PCV2 strains from different disease outbreaks in primary porcine monocytes: Stoon-1010 (PCV2a; PMWS), 1121 (PCV2a; abortion), 1147 (PCV2b; PDNS), 09V448 (PCV2d-1; PCVAD with high viral load in lymphoid tissues [PCVAD^high^]), DE222-13 (PCV2d-2; PCVAD^high^), and 19V245 (PCV2d-2; PCVAD^high^). The uptake of PCV2 in peripheral blood monocytes was different among the PCV2 strains. A large number of PCV2 particles were found in the monocytes for Stoon-1010, DE222-13, and 19V245, while a low number was found for 1121, 1147, and 09V448. Competition with, and removal of GAGs on the cell surface, demonstrated an important role of chondroitin sulfate (CS) and dermatan sulfate (DS) in PCV2 entry into monocytes. The mapping of positively/negatively charged amino acids exposed on the surface of PCV2 capsids revealed that their number and distribution could have an impact on the binding of the capsids to GAGs, and the internalization into monocytes. Based on the distribution of positively charged amino acids on PCV2 capsids, phosphacan was hypothesized, and further demonstrated, as an effective candidate to mediate virus attachment to, and internalization in, monocytes.

**IMPORTANCE** PCV2 is present on almost every pig farm in the world and is associated with a high number of diseases (PCV2-associated diseases [PCVADs]). It causes severe economic losses. Although vaccination is successfully applied in the field, there are still a lot of unanswered questions on the pathogenesis of PCV2 infections. This article reports on the uptake difference of various PCV2 strains by peripheral blood monocytes, and reveals the mechanism of the strong viral uptake ability of monocytes of Piétrain pigs. We further demonstrated that: (i) GAGs mediate the uptake of PCV2 particles by monocytes, (ii) positively charged three-wings-windmill-like amino acid patterns on the capsid outer surface are activating PCV2 uptake, and (iii) phosphacan is one of the potential candidates for PCV2 internalization. These results provide new insights into the mechanisms involved in PCVAD and contribute to a better understanding of PCV2 evolution. This may lead to the development of resistant pigs.

## INTRODUCTION

Porcine circovirus type 2 (PCV2) belongs to the circovirus genus of the *Circoviridae* family, and is the smallest virus that was found to infect mammals (1). PCV2 is the main etiology in porcine circovirus-associated diseases (PCVAD), combining several various syndromes, such as postweaning multisystemic wasting syndrome (PMWS), reproductive failure, porcine dermatitis, nephropathy syndrome (PDNS), respiratory, intestinal, and general diseases. It is an important pathogen that is economically challenging for the swine industry worldwide (2–5). As a result, most pigs are vaccinated early in their life.

PCV2 is a non-enveloped icosahedral virus, with a diameter of approximately 17 nm. The capsid encloses a single-stranded circular DNA genome with a length of 1767 to 1768 bp (6, 7). The PCV2 genome encodes three major open reading frames (ORFs). ORF1 encodes the replicase protein (Rep) which is associated with viral replication. ORF2 encodes the capsid protein (Cap), which is the sole structural protein. ORF3 is related to apoptosis and viral pathogenesis (8–11).

PCV2 replicates mainly in lymphoid tissues, which may result in histopathological lesions (lymphocyte depletion and monocyte infiltration) (12, 13). A variety of cells can be the target of PCV2 infection, but not all the cells support efficient viral replication. For a productive infection, the virus needs to hijack the cellular polymerase, which is mainly present in cells in mitosis. Consequently, proliferating lymphoblasts are important targets for PCV2; they efficiently produce new viruses. Large amounts of PCV2 antigens and nucleic acids can be found in infiltrating monocytes in lymphoid tissues, however, most monocytes are not productive (14, 15). Nevertheless, monocytes may harbor PCV2 antigens and genetic material for a long time. PCV2 has been demonstrated to be slowly internalized into epithelial cells, and cells of the monocytic cell line 3D4/31 through clathrin-mediated endocytosis (16, 17). Recent work from our lab also demonstrated that the entry of PCV2 into blood monocytes occurs via clathrin-mediated endocytosis with a partial degradation; the degree of PCV2 uptake and disintegration differed among pig breeds. Especially Piétrain pigs showed a stronger ability to take up viral particles compared to Landrace and Large White pigs (18).

Glycosaminoglycans (GAGs), including heparan sulfate (HS), heparin, chondroitin sulfate (CS), dermatan sulfate ([DS]; formerly called chondroitin sulfate B, CS-B), keratan sulfate (KS), and hyaluronan (HA), are heterogeneous, negatively charged, unbranched linear polysaccharides consisting of repeating disaccharide units that are usually covalently attached to various core proteins, forming proteoglycans (PGs) (19). Some GAGs can bind to a variety of pathogens, including viruses to promote attachment, invasion, and evasion of host defense mechanisms (20). Many viruses, such as PCV2, human papillomavirus (HPV), hepatitis B virus (HBV), and severe acute respiratory syndrome coronavirus 2 (SARS-CoV-2), have been reported to use GAG(s) as attachment receptors for the first unstable binding and transfer to other cellular receptors that mediate the internalization (21–23). PCV2 may use HS and CS to mediate the attachment onto 3D4/31 cells and T-lymphoblasts (24, 25). How PCV2 binds and enters monocytes has not been studied yet.

PCV2 has been isolated from pigs with different clinical pictures: PMWS, PDNS, abortion, and respiratory and intestinal problems. Furthermore, differences between isolates have been clearly demonstrated. As a result, it is important to figure out if there are strain differences that are responsible for the clinical outcome of an infection.

Therefore, this study aims to compare the difference in viral uptake by monocytes among different PCV2 strains, and to explain the differences from the perspective of virus-GAG interactions and amino acid distribution on the surface of PCV2 capsomers.

## RESULTS

### PCV2 uptake and disintegration kinetics in monocytes vary with virus strains.

**FIG 1:**
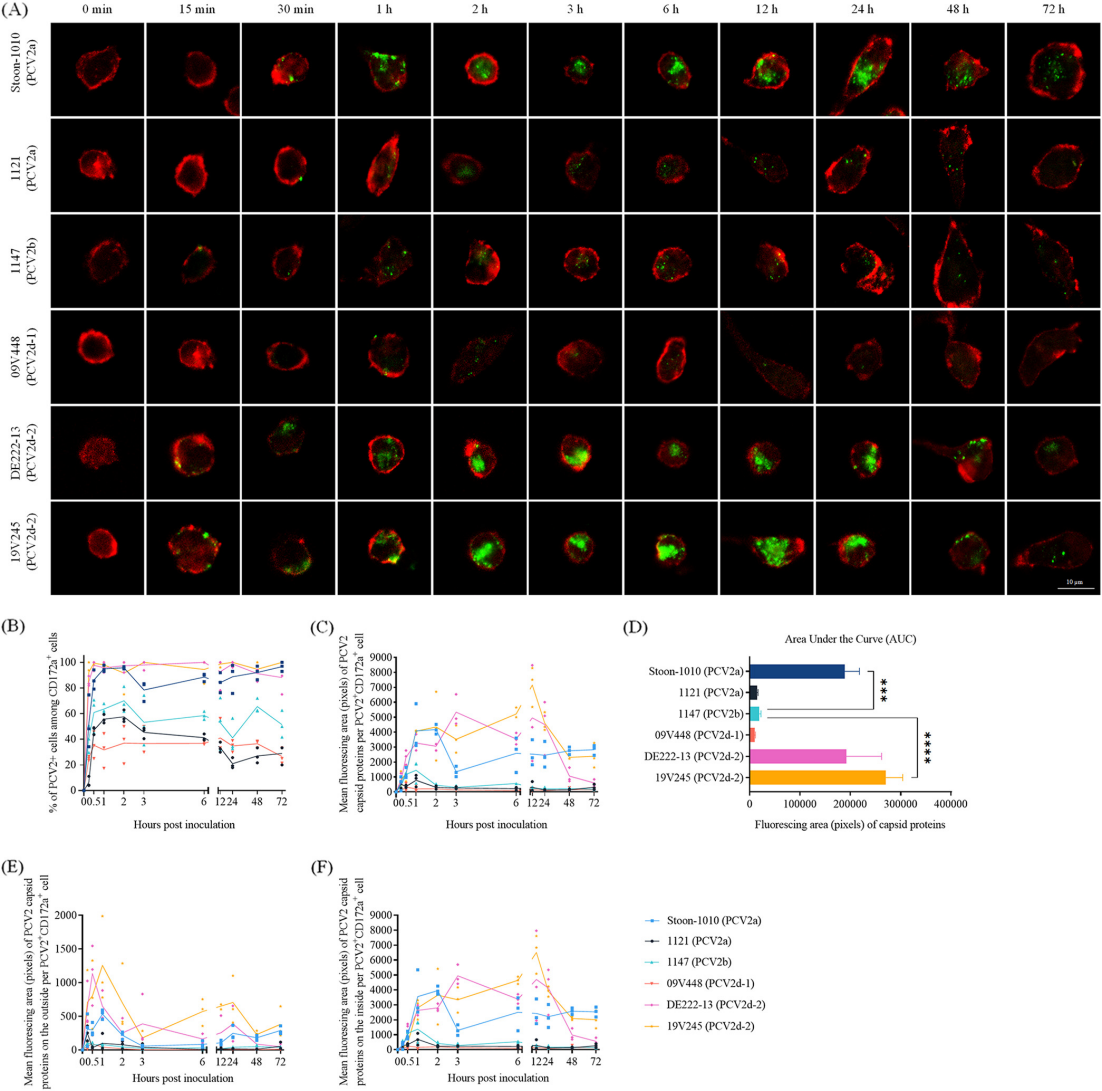
PCV2 uptake by peripheral blood monocytes of purebred Piétrain pigs in a strain-dependent manner. (A) Visualization of the PCV2 uptake by monocytes. PCV2 capsid proteins were stained in green, and monocytes were stained in red. Representative confocal images are given for each strain and each time point. (B to F) Analysis of the ability of blood monocytes to take up viral particles of different PCV2 strains. All data are the mean values of 3 replicates (3 pigs; each replicate is the mean of 10 randomly selected fields). (B) The percentage of PCV2 positive cells at each time point was calculated in 10 randomly selected fields. (C) The mean fluorescing area of capsid proteins in a PCV2 positive CD172a^+^ cell at each time point was calculated in the form of digital pixels with MATLAB. (D) The Area Under the Curve (AUC) of each strain was calculated. (E) Quantification of PCV2 particles that are attached to monocytes. The mean fluorescing area of viral capsids outside the cell was calculated. (F) Quantification of PCV2 particles that internalized into monocytes. The mean fluorescing area of viral capsids inside the cell was calculated. PCV2 particles were scored as internalized once they were inside the CD172a protein rim. Statistical difference (*P < *0.05) was demonstrated among 6 strains by one-way ANOVA with Turkey’s post-test. *, *P < *0.05; **, *P < *0.01; ***, *P < *0.001; ****, *P < *0.0001.

Previously, the monocytes from purebred and hybrid Piétrain pigs have been shown to have a more efficient ability to take up and disintegrate PCV2 capsids than purebred Large White and Landrace (18). Therefore, to investigate the difference in the uptake of PCV2 capsids among different PCV2 strains, purebred Piétrain pigs were used as blood donors in our study. The staining of viral quantification with red fluorescent microspheres is shown in Fig. S1. PCV2 uptake and disintegration are exhibited in Fig. 1. The strong PCV2 uptake ability of Piétrain monocytes is presented in Fig. 1A. The kinetics of the percentage of PCV2 positive cells differed among virus strains (Fig. 1B). Almost all monocytes were able to take up Stoon-1010, DE222-13, and 19V245 virions, while this ability with 1121, 1147, and 09V448 was only observed in approximately 40 70% of the monocytes. Over time, the percentage of monocytes with viral antigens inside the cytoplasm remained at a constant level for the strains Stoon-1010, DE222-13, 19V245, 1147, and 09V448, while 1121 significantly decreased with approximately 30%. At each time point, the amount of PCV2 particles that were taken up by monocytes and remained visible inside the cell was quantified in the form of digital pixels (Fig. 1C). The strains 1121, 1147, and 09V448 showed a low level uptake compared with the other 3 strains (Stoon-1010, DE222-13, 19V245); the number of incoming capsids peaked around 1 h in monocytes, then decreased to a low level at 3 h, and stayed at the same low level till the end of the experiment (72 h). Two to 13 times more Stoon-1010, DE222-13, and 19V245 particles were taken up than the former 3 strains during the first 3 h, then followed by a second increase at 6/12 h, and a steep drop at 48 and 72 h for the PCV2d strains DE222-13 and 19V245, whereas PCV2a strain Stoon-1010 remained at the same level. The area under the curve (AUC) of the fluorescing area of Cap uptake was also calculated for each strain (Fig. 1D), further supporting the stronger ability of Piétrain monocytes in taking up 19V245, DE222-13, and Stoon-1010.

The fluorescing area of capsids on the outside of the cell (attached to the cell surface) and the particles inside the cell (internalized by monocytes) was quantified (Fig. 1E and F). PCV2 virions quickly bound on the surface of monocytes within 1 h of incubation, and then efficiently became internalized. PCV2 19V245, DE222-13, and Stoon-1010 particles bound more to the cells than PCV2 1121, 1147, and 09V448 particles. Upon internalization, a large proportion of Cap proteins of 1147, DE222-13, 19V245, and 1121 were destroyed at 72 h at a level of 89%, 89%, 70%, and 63%, respectively, while less than 50% of internalized capsids were degraded with 09V448 and Stoon-1010, which indicated the weak ability of monocytes to decompose the capsids of the latter 2 strains.

### Expression of HS, CS-A/C, and decorin (indication of DS) on monocytes.

**FIG 2:**
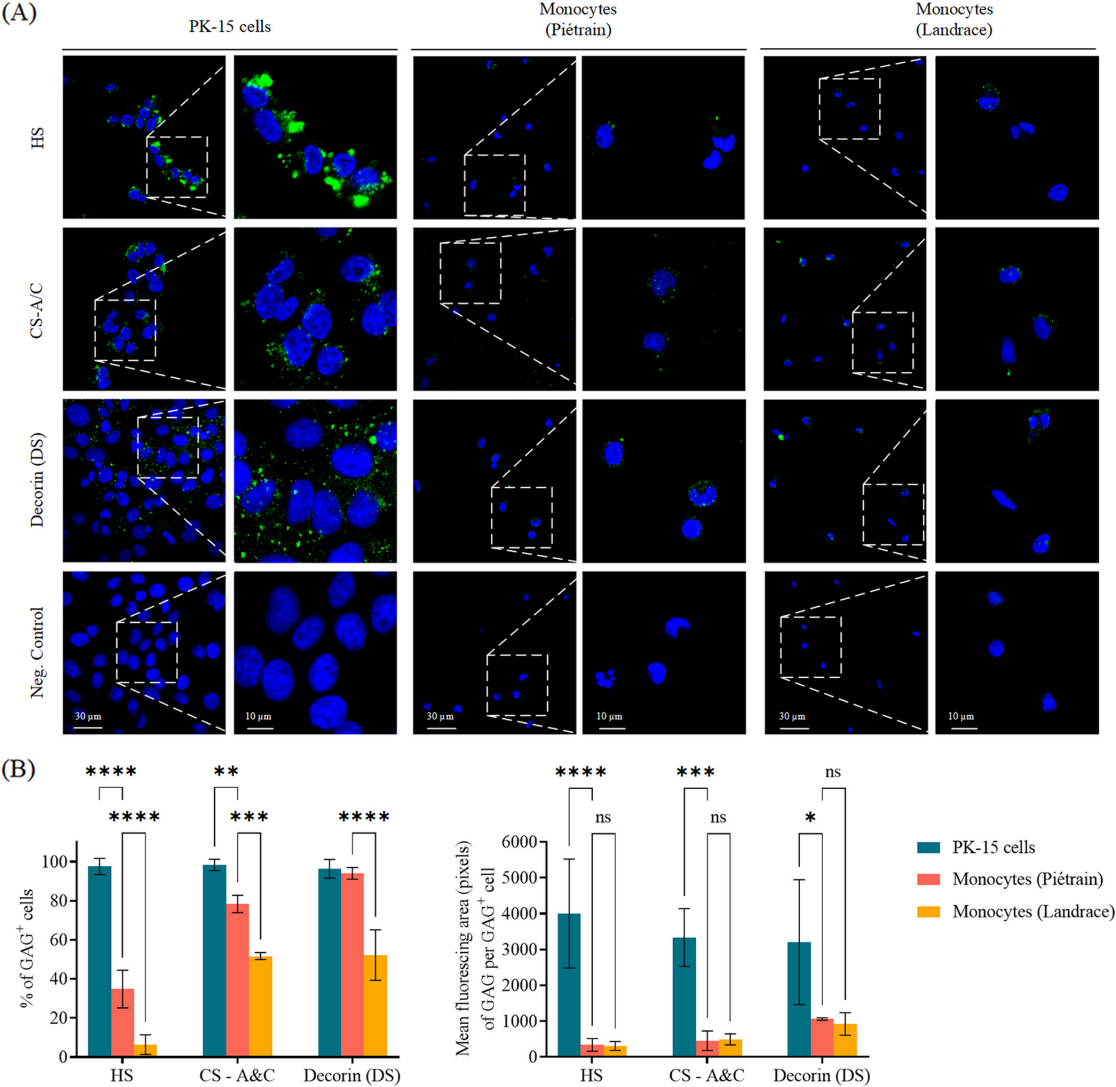
Expression of heparan sulfate (HS), chondroitin sulfate (CS), and decorin (linked with dermatan sulfate [DS] chains) on PK15 cells and peripheral blood monocytes of purebred Piétrain and Landrace pigs. Data were obtained from 3 different wells for PK-15 cells, and 3 pigs of each breed for monocytes. (A) Confocal images showing PK15 cells and peripheral blood monocytes stained with specific antibodies against HS, CS, and decorin. (B) Quantification of the percentage of PK-15 cells and monocytes expressing HS, CS, decorin, and the mean fluorescing area (pixels) of GAG per GAG^+^ cell. Data represent the means ± SD of 3 experiments. Differences between PK-15 cells and monocytes were revealed by two-way ANOVA with Dunnett’s post-test. **, *P < *0.01; ***, *P < *0.001; ****, *P < *0.0001.

PCV2 has been proven to use HS and CS-B (DS) as PCV2 attachment receptors on cells of the porcine monocytic cell line 3D4/31, and the porcine kidney epithelial cell line PK-15 (24). CS (and not HS) has also been demonstrated to be expressed, and used, as a receptor on/in peripheral blood T-lymphoblasts (18). To determine whether HS, CS, and CS-B (DS) are expressed on the surface of monocytes from Piétrain and Landrace pigs, specific antibodies were used in this experiment. PK-15 cells were used as a positive control due to the high expression of HS, CS and decorin (the protein core linked with DS). Figure 2 shows that almost all PK15 cells exhibited a strong expression of HS, CS, and decorin. The expression of each GAG on monocytes was generally lower than on PK-15 cells. When comparing the 2 breeds, the expression of HS, CS, and decorin was observed on 34.8%, 78.4%, and 94.0% of the cells, respectively, in Piétrain pigs, and on 6.3%, 51.7%, and 52.2% of the cells in Landrace pigs. Per GAG^+^ cell, HS, CS, and decorin (DS) were highly expressed on PK-15 cells; there was no significant difference in GAG expression between monocytes from Piétrain and monocytes from Landrace pigs.

### PCV2 particles efficiently bind to monocytes with CS-A/C and decorin (DS), but weakly to monocytes with HS.

**FIG 3:**
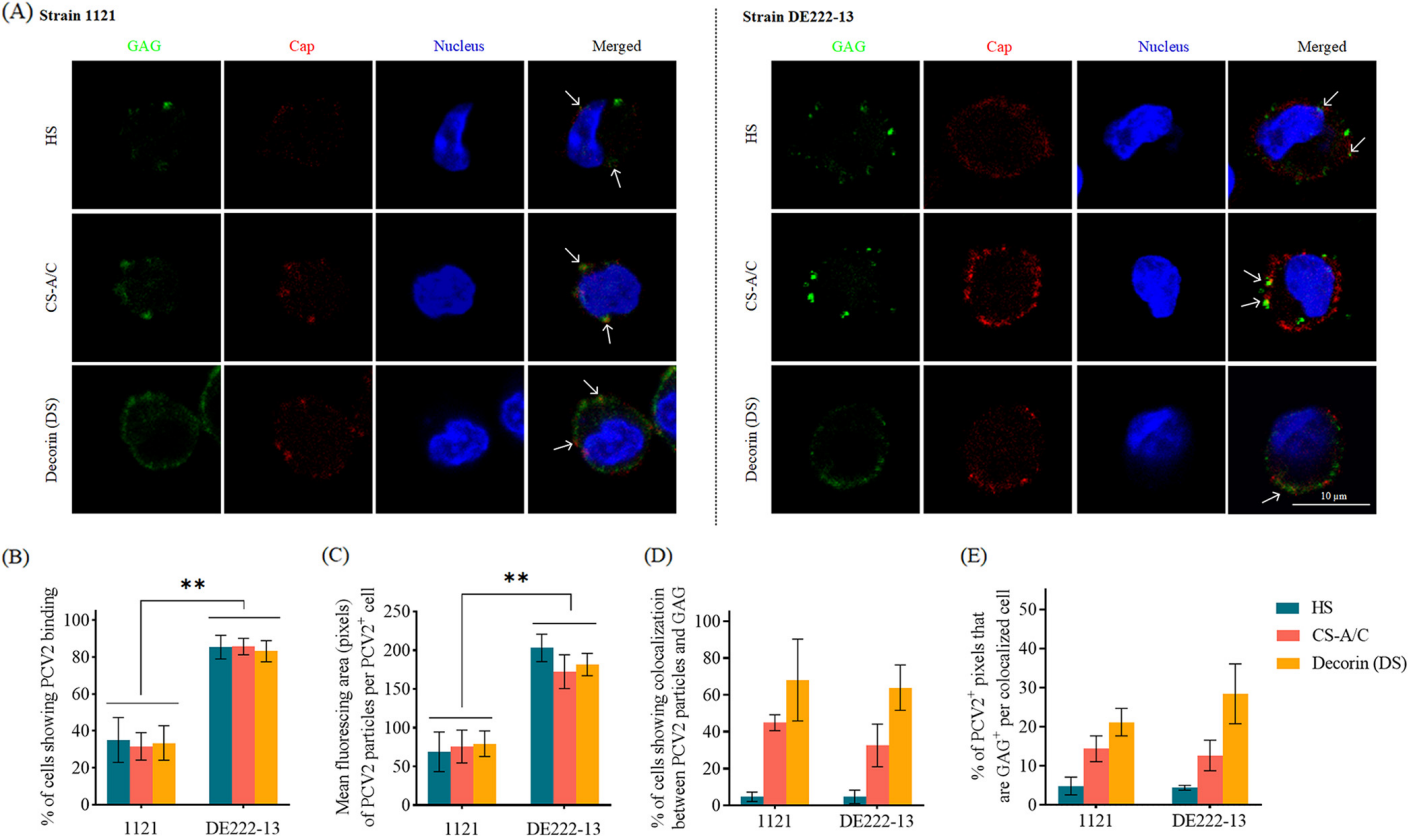
Colocalization of GAG (HS, CS-A/C, decorin [DS]) and PCV2 particles. Blood monocytes from 3 Piétrain pigs were incubated with PCV2 particles at 4°C for 1 h, after which the virus was removed, and cells were fixed with 4% PF. Double immunofluorescence staining to detect GAG and PCV2 capsid proteins was performed. GAG was stained in green, and the PCV2 virions were stained in red. Cell nuclei were co-stained in blue. Visualization of colocalization was performed by confocal microscopy. White arrows indicate the colocalization between GAG and PCV2 particles in merged images. All data are the mean values of 3 replicates (3 pigs; each replicate is the mean of 10 randomly selected fields). (A) Representative pictures of cells with 1121 and DE222-13 PCV2 particles colocalized with GAG. (B) The percentage of PCV2-antigen-positive cells was determined. (C) Fluorescing area of attached PCV2 particles per cell was quantified by ImageJ in pixels. (D) The percentage of cells showing colocalization between PCV2 particles and GAG was determined. (E) For each PCV2-GAG colocalized cell, the proportion of PCV2 positive pixels that are also GAG positive was assessed based on individual fluorescing pixels per cell. Data represent the means ± SD of 3 experiments. Differences between 1121 and DE222-13 were revealed by the Student's *t* test. **, *P < *0.01.

The attachment experiment followed by a double immunofluorescence staining was performed to investigate whether PCV2 binding to monocytes is happening through GAG molecules. Each GAG was stained with different specific antibodies, and PCV2 was stained with mouse anti-Cap IgG2b 12E12 MAb (Fig. 3A). Particles of 1121 and DE222-13 bound to 33.4% and 84.7%, respectively, of the total monocytes (Fig. 3B). The fluorescence intensity of DE222-13 Caps on monocytes was also higher than that of 1121 (Fig. 3C). It indicated a stronger binding ability of DE222-13 compared to that of 1121, which is in line with the uptake difference in Fig. 1.

The colocalization between PCV2 particles and GAG was examined by counting the percentage of cells, showing PCV2-GAG colocalization among the total PCV2 positive cells, and quantifying the PCV2-positive digital pixels that were also GAG positive per PCV2 positive cell.

Of the cells with attached 1121 and DE222-13 particles, different GAGs showed different colocalization efficiency with PCV2 Caps. It exhibited the lowest, moderate, and highest colocalization between PCV2 particles and HS, CS-A/C, and decorin (DS), respectively. Specifically: (i) 4.7% of PCV2 (1121 and DE222-13) positive cells were also positive for HS, (ii) 44.9% (1121) and 32.6% (DE222-13) of PCV2 positive cells were also positive for CS-A/C, and (iii) approximately 65% PCV2 (1121 and DE222-13) cells were also positive for decorin (Fig. 3D). To evaluate the colocalization ratio per PCV2-GAG colocalized cell, the percentage of pixels that were both PCV2-GAG positive (colocalization) on the total of PCV2 positive pixels was determined (Fig. 3E). From all PCV2 positive pixels, 4.8% (1121) and 4.4% (DE222-13) colocalized with HS, 14.4% (1121) and 12.6% (DE222-13) colocalized with CS-A/C, and 21.2% (1121) and 28.4% (DE222-13) colocalized with decorin.

Taken together, the results suggest that PCV2 binds mainly with DS (by testing decorin) and CS-A/C on monocytes, but restrictedly with HS; strain DE222-13 is more efficient to bind to monocytes than 1121. Further, the results demonstrated that also other target molecules are used for binding to monocytes.

### GAGs mediate PCV2 attachment to monocytes, and play an important role in PCV2 internalization.

**FIG 4:**
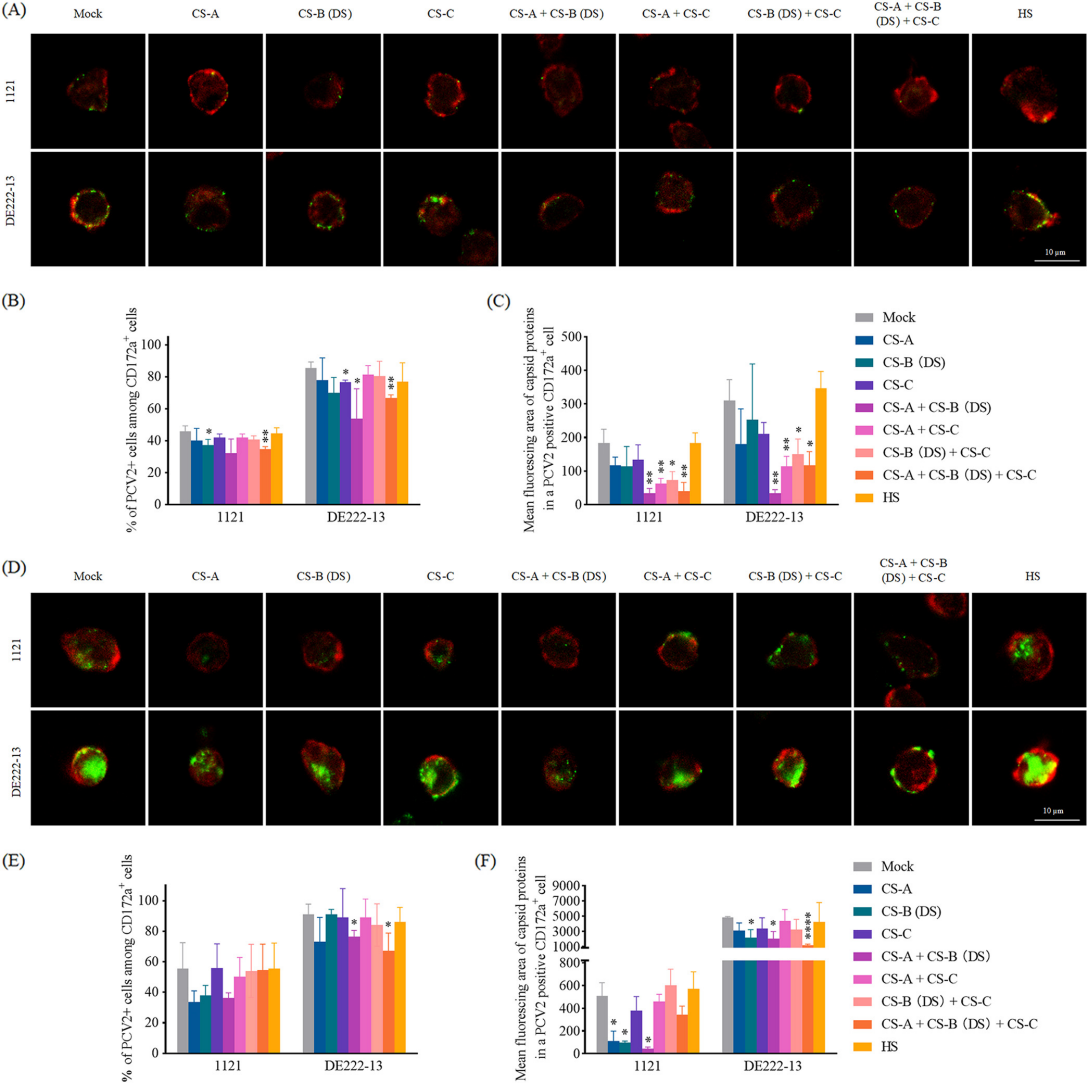
Effects of GAGs on virus attachment (A, B, and C) and internalization (D, E, and F) on/in blood monocytes from 3 Piétrain pigs. Equal amounts of PCV2 particles were mixed with individual GAGs or GAG combinations at 37°C for 90 min before being added to monocytes for an incubation period of 1 h at either 4°C or 37°C. For confocal microscopy, 10 random fields of each slide/each pig were selected for quantification. (A and D) Visualization of PCV2 particles attached to the monocyte surface (A)/internalized into monocytes (D) was conducted by LSCM. Bar = 10 μm. (B and E) The percentage of monocytes with PCV2 sticking on the cell surface (B)/internalization into the cell (E) was counted. (C and F) The fluorescing area of attached (C)/internalized (F) viral particles per cell was quantified with MATLAB. Data represent the means ± SD of 3 independent experiments (3 pigs). For each strain, every bar was compared with its Mock, and the significance of the differences was calculated by the Student's *t* test. *, *P < *0.05; **, *P < *0.01; ****, *P < *0.0001.

Some GAGs have been proven to be important receptors for cell adhesion and internalization to mediate infection of many viruses (26). To bring additional proof of the role of GAGs in PCV2 uptake of monocytes, a competition experiment was performed. A mixture of PCV2 and GAG(s) was added to monocytes for a 1 h incubation, at either 4°C for attachment or 37°C for internalization. The percentage of PCV2 positive cells and Cap fluorescing area per PCV2 positive cell was calculated. In the mock-treated monocytes, strain 1121 was detected at a lower level than DE222-13, revealing its relatively inefficient binding to monocytes. As shown in Fig. 4B and 4C, HS has little effect on PCV2 binding; CS moderately reduced the adhesion of virus particles. Additive effects were detected in different combinations with the different CS. Especially, when CS-A was mixed with either CS-B (DS) or CS-C before being added to the cells, the viral attachment dramatically dropped by 90% and 70%, respectively. Combining CS-B with CS-C had a lower effect. Therefore, GAGs may mediate PCV2 attachment, especially in combination.

In the mock-treated control, strain 1121 showed again a weaker internalization than DE222-13 (Fig. 4D to F). HS is barely involved in the PCV2 internalization. CS-C weakly interfered in this process. For individual GAG, CS-A and CS-B (DS) clearly reduced the internalization of viral particles, especially with CS-B (DS) showing the strongest power. For the combinations, only CS-A + CS-B (DS) and CS-A + CS-B (DS) + CS-C had an obvious extra decline compared to the individuals. Other combinations with CS-C seem to have an opposite effect. They abolished the block caused by the other GAGs. Taking all the results together, it was shown that CS-A, CS-B (DS) and CS-C are all relevant to PCV2 attachment on the cell surface, and help each other in the viral binding process, whereas mainly CS-A and CS-B (DS) mediate PCV2 internalization in monocytes; CS-C had an activating effect.

### Enzymatic removal of CS-A/B/C reduced PCV2 uptake on monocytes.

**FIG 5:**
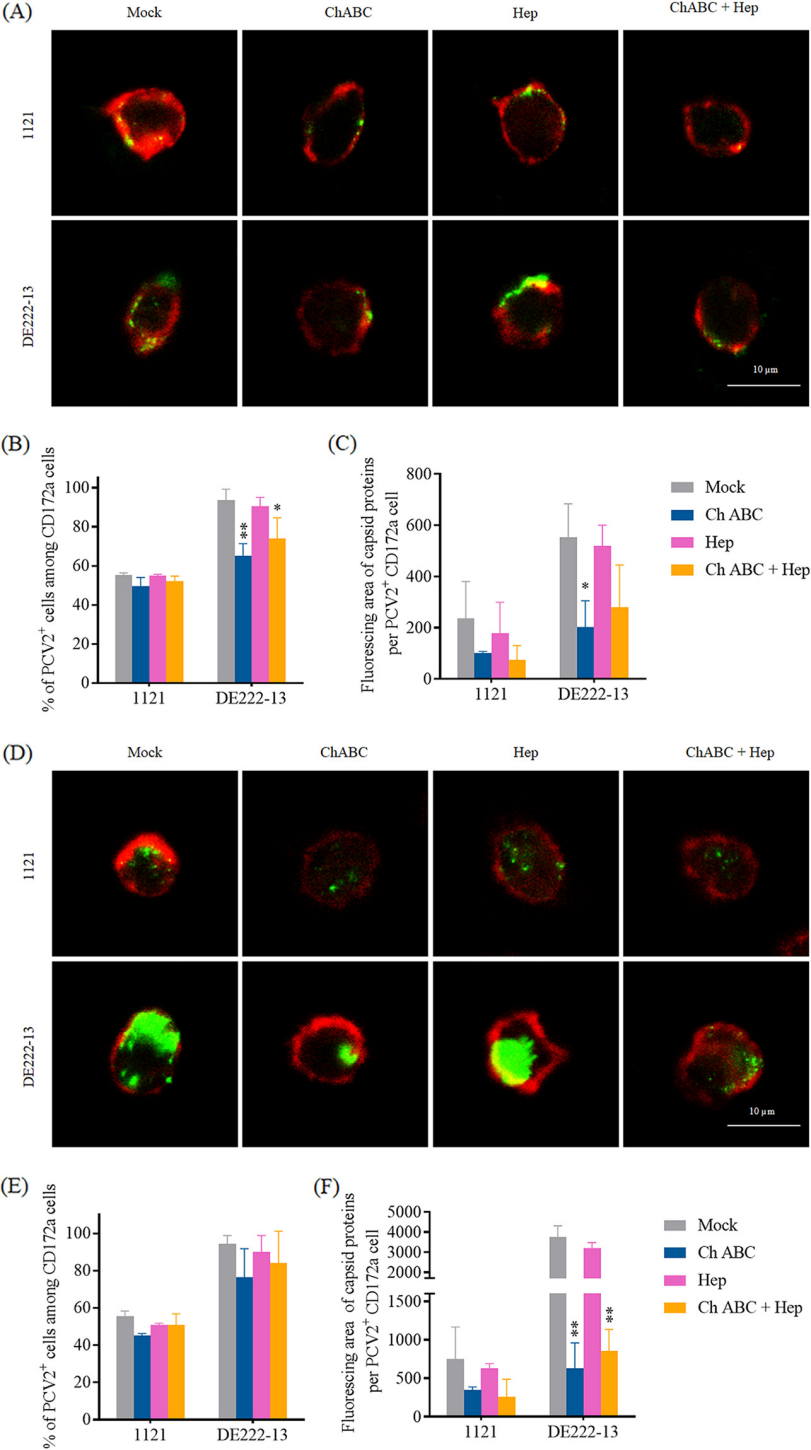
Effects of enzymatic removal of CS-A/B/C and/or HS on PCV2 attachment (A, B, and C) onto the surface of blood monocytes, and on the PCV2 internalization (D, E, and F) into these cells. Cells were isolated from 3 purebred Piétrain pigs, and pretreated with either chondroitinase ABC or heparinase I&III, or the combination of the 2 enzymes at 37°C for a 1-h treatment before virus incubation at either 4°C or 37°C for 1 h. For confocal microscopy, 10 random fields of each slide/each pig were selected for quantification. (A and D) Visualization of PCV2 attachment onto (A)/internalization into (D) monocytes. Bar = 10 μm. (B and E) The percentage of cells showing PCV2 attachment (B)/internalization (E) was determined. (C and F) The fluorescing area of attached (C)/internalized (F) virus particles per cell was quantified with MATLAB. Data represent the means ± SD of 3 experiments (3 pigs). For each strain, every bar was compared with its Mock, and the significance of the differences was estimated by Student's *t* test. *, *P < *0.05; **, *P < *0.01.

To further investigate the role of CS-A, CS-B (DS) and CS-C on PCV2 uptake of monocytes, cells were pre-incubated with either chondroitinase ABC (ChABC), Heparinase I&III (Hep), or the combination of ChABC and Hep before incubating with PCV2, to cleave the GAG chains from the cell surface. In this experiment, viruses were inoculated at either 4°C for attachment or 37°C for internalization. Pretreatment with ChABC significantly decreased the viral binding and internalization, while this did not happen with the Hep treatment group (Fig. 5). Consequently, a clear role of CS-A/B/C has been demonstrated in the PCV2 uptake by peripheral blood monocytes.

### Structural differences in capsid proteins among the six PCV2 strains may explain the difference in viral uptake by monocytes.

**FIG 6:**
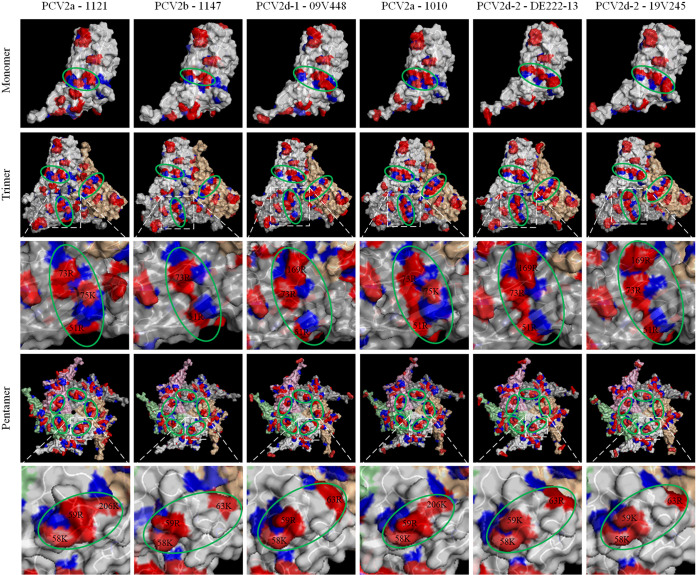
Three dimensional mapping of the monomer, trimer, and pentamer of the PCV2 capsid protein of the PCV2 strains PCV2a (Stoon-1010), PCV2a (1121), PCV2b (1147), PCV2d-1 (09V448), PCV2d-2 (DE222-13), and PCV2d-2 (19V245). Charged residues were labeled in 2 major colors: positively charged residues (arginine [R], lysine [K], histidine [H])-red; negatively charged residues (aspartic acid [D], glutamic acid [E]-blue. The upper row presents the outer surface of the PCV2 capsid monomer. The middle rows present the outer surface of the PCV2 capsid trimer and the magnification of 1 of the positively charged bands of the three-wings-windmill-like pattern. Two configurations were found based on the specific positively charged bands in the middle of these models (circled in green). Pattern 1 shows an incompact and disconnected pattern, while pattern 2 shows a connected and regular three-wing-windmill pattern. The lower rows present the outer surface of the PCV2 capsid pentamer, and the magnification of 1 of the positively charged patches of the pentagon pattern. The amino acids composing the pentagon were circled in green. The 3D structures of monomeric capsid were predicted using the I-TASSER server. The trimeric and pentameric subunits were generated by SYMMDOCK server. All models here were displayed with PyMOL.

Positively/negatively charged amino acids that are exposed on the PCV2 capsid surface could be important for the initial attachment of the virus to host cells, due to the interaction with the negatively charged GAGs. The alignment of the amino acid sequences from the six PCV2 strains showed a variation of amino acids as displayed in Fig. S2. The N termini of the PCV2 Cap proteins of the 6 strains are very conserved and contain many positively charged amino acids (aa) Arg (R), Lys (K), and His (H). It is predicted that this region does not have a secondary structure, is interacting with the negatively charged viral genome, and is not involved in defining the three-dimensional (3D) structure of the Cap protein (27, 28). Thus, the N-terminus peptide (1 to 42 aa) of each PCV2 Cap sequence was not displayed in these structures. Notably, an extra residue of 234K/R was present at the end of the C-terminus of the PCV2d strains. The positive and negative charges on the capsid surface were displayed in monomers, trimers, and pentamers (Fig. 6). For the monomeric capsid, there is an obvious enrichment of several positively charged amino acids (51R, 73R, 75K, 169R) in the middle of the surface (encircled with a green dashed line). For Stoon-1010, DE222-13, and 19V245, these amino acids are forming a complete positively charged band, while for the 3 strains 1121, 1147, and 09V448, this band is discontinuous and seems to fail to line up. From the perspective of the capsid trimer, the 3 positively charged bands displayed an interesting counterclockwise three-wings-windmill-like pattern. From the perspective of the capsid pentamer, another positive band of positively charged amino acids showed up for the PCV2a strains 1121 and Stoon-1010 on the monomer that formed a pentagon in the pentamer configuration, while it only displayed 5 dispersed patches for the PCV2b and PCV2d strains (Fig. 6). When combining the trimer and pentameric data, it can be concluded that the low-PCV2-binding/internalizing strains 1121 (PCV2a), 1147 (PCV2b), and 09V448 (PCV2d-1) did not exhibit one of the patterns (1147 & 09V448), or only the pentagon pattern (1121). The high-PCV2 binding/internalizing strains Stoon-1010 (PCV2a), DE222-13 (PCV2d-2), and 19V245 (PCV2d-2) contained either both patterns (Stoon-1010), or only the three-wings-windmill pattern (DE222-13 and 19V245).

Data were additionally analyzed by PyMOL software. The trimer and pentamer surfaces of each PCV2 strain had the same position and number of exposed positive/negative residues, and the solvent-accessible surface area (SASA) of the 2 different subunits was highly similar. Data were presented in Table 1 based on the trimeric configuration. According to Table 1, Stoon-1010, DE222-13, and 19V245 have 2 more positively charged residues than negatively charged residues, 1121 and 09V448 have 1 more positively charged residue than negatively charged residues, whereas 1147 have equal numbers of positively and negatively charged residues. This implies that Stoon-1010, DE222-13, and 19V245 bind to GAGs on the cell surface more efficiently than 1121, 09V448, and 1147. The SASA of positively and negatively charged residues was also calculated. Compared with 1121 and 1147, a more positive ΔSASA was displayed on the outside surface of the capsid of Stoon-1010, DE222-13, 09V448, and 19V245.

**TABLE 1:** Data on amino acids with positive/negative charges exposed on the outer capsid surface (per monomer; based on trimer configuration)

Genotype	Strain	Position of positive (+) residues	Position of negative (−) residues	No. of positive (+) residues	No. of negative (−) residues	ΔResidues^*a*^	SASA^*b*^ (+)(Å^2^)	SASA (−)(Å^2^)	ΔSASA^*c*^ (Å^2^)
PCV2a	Stoon-1010	51R, 58K, 59R, 73R, 75K, 88K, 132K, 186R, 206K, 227K, 232K	70D, 77D, 78D, 115D, 127D, 168D, 194D, 208D, 210D	11	9	2	1050.50	270.90	779.60
PCV2a	1121	51R, 58K, 59R, 73R, 75K, 88K, 132K, 186R, 191R, 206K, 227K	70D, 77D, 78D, 115D, 126D, 127D, 168D, 194D, 208D, 210D	11	10	1	1143.64	458.33	685.31
PCV2b	1147	51R, 58K, 59R, 63K, 73R, 89R, 132K, 186R, 227K	70D, 78D, 115D, 126D, 127D, 168D, 194D, 208D, 210E	9	9	0	785.88	267.04	518.84
PCV2d-1	09V448	51R, 58K, 59R, 63R, 73R, 132K, 169R, 186R, 227K, 234K	70D, 78D, 115D, 126D, 127D, 168D, 194D, 208D, 210D	10	9	1	1166.70	294.83	871.87
PCV2d-2	DE222-13	51R, 58K, 59K, 63R, 73R, 132K, 169R, 186R, 227K, 234R	70D, 78D, 115D, 127D, 168D, 194D, 208D, 210D	10	8	2	1123.97	288.93	835.04
PCV2d-2	19V245	51R, 58K, 59K, 63R, 73R, 132K, 169R, 186R, 227K, 234K	70D, 78D, 115D, 127D, 168D, 194D, 208D, 210D	10	8	2	1003.89	268.74	735.15

a ΔResidues = number of positive (+) residues − number of negative (−) residues (per monomer).

b SASA: solvent-accessible surface area (per monomer).

c ΔSASA = SASA (+) − SASA (−) (per monomer).

### PCV2 uptake by monocytes could be mediated by phosphacan.

**FIG 7:**
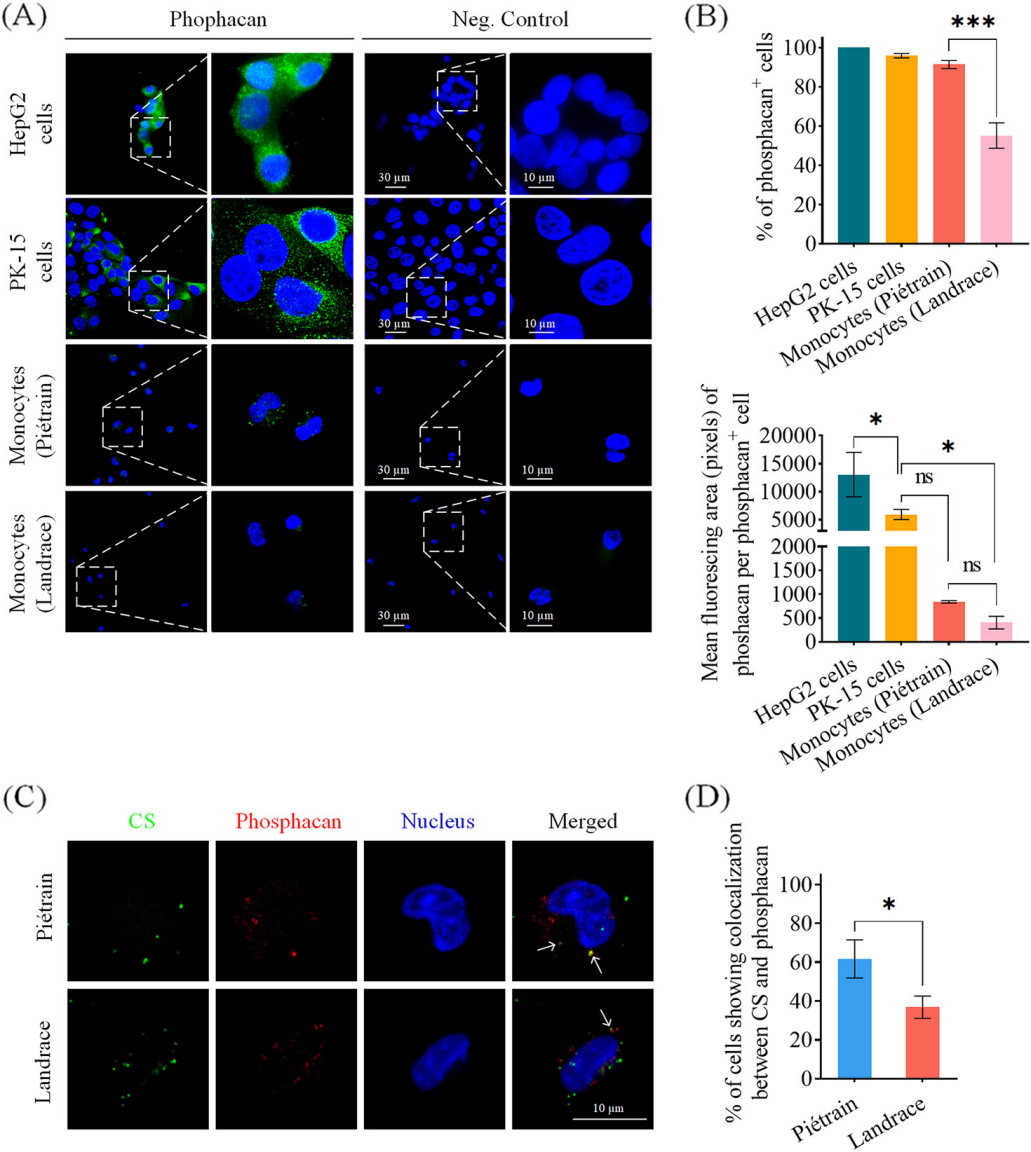
Expression of phosphacan on HepG2 cells (positive control), PK-15 cells, and peripheral blood monocytes of Piétrain and Landrace pigs, and colocalization of CS and phosphacan. Monocytes were fixed with 4% PF at RT for 10 min, followed by double immunofluorescence staining with specific primary antibodies and conjugated secondary antibodies. (A) Confocal images showing HepG2 cells, PK-15 cells, and monocytes stained with a specific antibody against phosphacan. (B) Quantification of the percentage of cells expressing phosphacan, and the mean fluorescing area (pixels) of phosphacan per phosphacan^+^ cell. (C) Representative images showing colocalization of CS and phosphacan on monocytes. White arrows indicate the colocalization between CS and phosphacan in merged images. (D) Quantification of the percentage of cells showing colocalization of CS and phosphacan. Data represent the means ± SD of 3 experiments. Differences between Piétrain and Landrace were revealed by the Student's *t* test. *, *P < *0.05; ***, *P < *0.001.

Based on the three-wings-windmill-shaped positively charged bands of PCV2 capsids, phosphacan could serve as an important transmembrane molecule to mediate the uptake of PCV2 virions by monocytes due to its multiple attachment sites of CS chains (can be sporadically substituted with KS chains). The 3 prominent bands of positively charged amino acids in the 3D structure (trimer) of PCV2 capsids could bind with any three CS chains of phosphacan, and be endocytosed by monocytes. Therefore, the expression of phosphacan in PK-15 cells and monocytes from Piétrain and Landrace pigs was examined by immunofluorescence staining with the specific rabbit anti-PTPRZ IgG pAb (Fig. 7A). HepG2 cells were used as a positive control in this experiment. Almost all PK-15 cells were phosphacan positive. Also, 91% of monocytes from Piétrain pigs expressed phosphacan. In contrast, only 55% was observed in monocytes isolated from Landrace pigs. Per phosphacan^+^ cell, phosphacan was highly expressed on HepG2 cells and PK-15 cells; there was no significant difference in GAG expression between monocytes from Piétrain and monocytes from Landrace pigs (Fig. 7B).

The colocalization of CS and phosphacan was also investigated to confirm their link by a double immunofluorescence staining (Fig. 7C). Compared with Landrace pigs (36.8%), the monocytes from Piétrain pigs (61.7%) exhibited a higher percentage of colocalization between CS and phosphacan (Fig. 7D).

**FIG 8:**
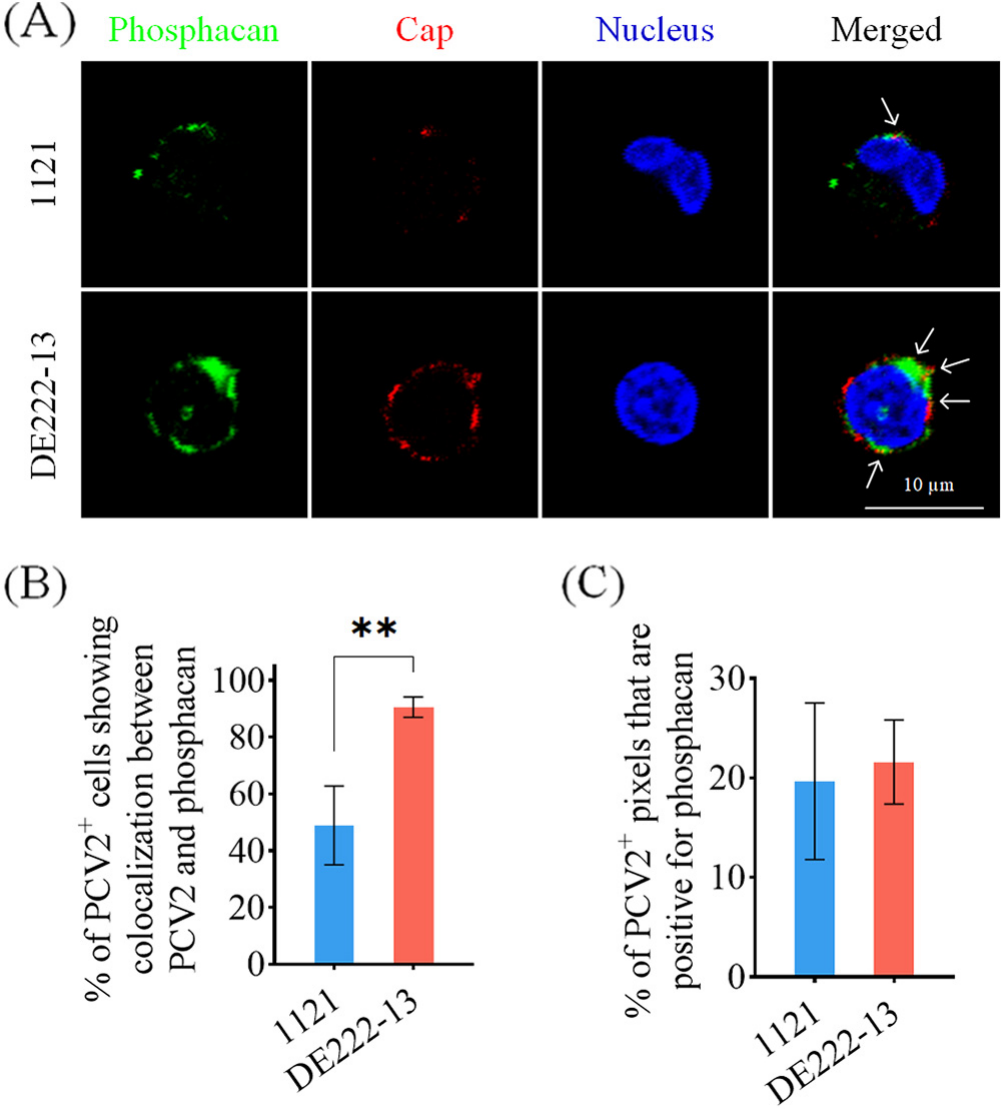
Colocalization of PCV2 and phosphacan. Monocytes were incubated with PCV2 particles at 4°C for 1 h, after which the virus was removed and cells were fixed with 4% PF. Phosphacan was stained in green, and PCV2 particles were stained in red. Cell nuclei were co-stained in blue. (A) Representative pictures of cells with 1121 and DE222-13 particles showing colocalization with phosphacan. White arrows indicate the colocalization between PCV2 virions, and phosphacan in merged images. (B) The percentage of cells showing colocalization between PCV2 particles and phosphacan was determined. (C) For each PCV2-phosphacan colocalized cell, the proportion of PCV2 positive pixels that are also phosphacan positive was quantified based on their individual fluorescing pixels per cell. Data represent the means ± SD of 3 experiments. Differences between 1121 and DE222-13 were revealed by the Student's *t* test. **, *P < *0.01.

Similar to the assay of PCV2-GAG colocalization, another double immunofluorescence staining was performed to reveal the colocalization between PCV2 particles and phosphacan with the antibodies specifically targeting phosphacan and PCV2 Caps. Of the cells with bound 1121 or DE222-13 particles, 48.9% versus 90.6% were phosphacan positive (Fig. 8B). For estimating the colocalization ratio per PCV2-phosphacan colocalized cell, the percentage of PCV2 positive pixels that were also phosphacan positive was calculated. Both strains exhibited a similar ratio of about 20% in colocalization with phosphacan (Fig. 8C).

## DISCUSSION

Virus entry into cells is initiated by the attachment to receptors (29). GAGs, which are widely distributed on the cell membrane and extracellular matrix, have been shown to be important attachment receptors for many viruses (24, 30–32).

In this study, it was shown that negatively charged GAGs (mainly CS and DS, but not HS) mediate the binding of PCV2 virions to the monocyte cell surface, facilitating viral uptake. This was demonstrated by GAG competition experiments, enzymatic removal of GAGs and colocalization studies. The strain-dependent uptake by monocytes could be explained by the net charges that are exposed on the outside of the virus, together with the distribution pattern of the charged amino acids. Indeed, the strains with at least 2 more positively charged amino acids than negatively charged amino acids on the surface (PCV2a-1010 [*n* = 2], PCV2d-2-DE222-13 [*n* = 2], PCV2d-2-19V245 [*n* = 2]) were more easily taken up by monocytes than strains with almost equal number of positively and negatively charged amino acids (PCV2a-1121 [*n* = 1], PCV2b-1147 [*n* = 0] and PCV2d-1-09448 [*n* = 1]). In addition, for the 3 strains PCV2a-1010, PCV2d-2-DE222-13, and PCV2d-2-19V245, the distribution of the positively and negatively charged residues was arranged in a specific way. In the middle of the capsid monomers, a line of positively charged arginines was positioned on top of some negatively charged aspartic acids, which were located in the background. Looking at capsid trimers, the 3 lines formed a special structure of a three-wings-windmill. Additionally, spots of positively charged lysines were found outside the wings (Fig. 6). With the capsid pentamers (centered on the 5-fold axis) of the PCV2a strains 1121 and 1010, an extra intriguing pentagon pattern was found with 3 positively charged residues (58K, 59R/K, 63R/K, or 206K) on the top of the outer surface of each monomeric subunit. The other strains did not demonstrate this pentagon symmetry. It is now hypothesized that the combination of the three-wings-windmill and the pentagon are responsible for the PCV2 binding to GAGs. This may explain why strains PCV2b 1147 and PCV2d-1 09V448 (no three-wings-windmill; no pentagon) did not attach to monocytes as effectively as Stoon-1010 (both three-wings-windmill and pentagon are present) and the PCV2d-2 strains (three-wings-windmill present; no pentagon). The PCV2a 1121 strain showed only the pentagon pattern (no three-wings-windmill), suggesting that the pentagon pattern has a lower avidity toward the GAGs (Table 2).

**TABLE 2:** Composition of amino acids displayed on the three-wings-windmill/pentagon pattern of a PCV2 capsid trimer/pentamer

Genotype	Strain	Trimer		Pentamer	
		Three-wings-windmill^*a*^	Involved AAs^*b*^	Pentagon^*a*^	Involved AAs^*b*^
PCV2a	Stoon-1010	+	51R/73R/75K	+	58K/59R/206K
PCV2a	1121	−	51R × 73R/75K	+	58K/59R/206K
PCV2b	1147	−	51R × 73R	−	58K/59R × 63K
PCV2d-1	09V448	−	51R × 73R/169R	−	58K/59R × 63R
PCV2d-2	DE222-13	+	51R/73R/169R	−	58K/59K × 63R
PCV2d-2	19V245	+	51R/73R/169R	−	58K/59K × 63R

a The three-wings-windmill pattern in the trimer or pentagon pattern in the pentamer are present (+) or absent (−).

b The positively charged amino acids are spatially connected (/) or not connected (×).

The viruses that originated from PCVAD cases with high viral loads in lymph nodes (PMWS-like syndrome) were strongly taken up by monocytes (except for the PCV2-d1-09V448). This fits with the fact that monocytes, which are massively taking up PCV2 adversely, modulate the immune system. McCullough et al. (33, 34) demonstrated that, although PMWS is associated with lymphopenia and a general collapse of the immune system, this is not a direct outcome of the virus’s interaction with lymphocytes. It was speculated that overloaded monocytes were key to this phenomenon. Further work is needed to understand the effect of overloading monocytes with PCV2 particles and other pathophysiological effects in PMWS pigs, such as lymphocyte loss.

Table S1 compares the effects of different GAGs on PCV2 uptake using 2 different technologies (GAG competition and enzymatic GAG removal). It became clear that, although DE222-13 (PCV2d-2) is in general a stronger internalizer than 1121, both 1121 (PCV2a) and DE222-13 use CS-A/C and decorin (CS-B/DS) as main entry mediators. In the Table S1, it can be seen that adding CS-A, CS-B (DS), and CS-C to the virus all caused an average drop rate of around 30% on PCV2 pixels for attachment, and it was further reduced by up to 80% when CS-A and CS-B (DS) were used together, demonstrating that CS-A and CS-B (DS) had additive and high-efficiency impacts on PCV2 binding. The combined use of CS-A+CS-B(DS)+CS-C mitigated this decrease, possibly due to the cross-linking of CS-C with CS-A and/or CS-B(DS), which may have lowered the efficiency of the CS-A+CS-B(DS)-mediated action (35). Like attachment, different GAGs (and combinations) decreased PCV2 internalization to different degrees. When GAGs were separately used, CS-B (DS) caused the highest reduction (over 50%) for both strains, followed by CS-A (±25%) and CS-C (±10–20%), suggesting the major role of CS-B (DS) in mediating virus internalization. Combining CS-A with CS-B(DS) had an additive effect on the internalization for 1121, but not for DE222-13. Also in these experiments, CS-C showed negative effects in combinations. Although all examined GAGs (CS-A, CS-B, CS-C) contribute equally to the attachment, mainly CS-B is involved in the internalization. The use of the enzyme ChABC (cleaves CS-A, B, and C) provided additional evidence of the importance of GAGs in PCV2 binding and internalization. Pixel drop rates between ChABC (enzymatic removal of GAGs) and CS-A+CS-B(DS)+CS-C (addition of GAGs) are comparable, indicating that PCV2 uptake is achieved through CS-A/B/C.

The type and chain length of GAGs may determine their biological functions. Moreover, the degree and position of sulfation and the extraction technology may also have an impact on the behavior of the polymer chains. The powerful negative charge of GAGs is imparted by sulfate and/or carboxyl groups at various positions on the GAG chains (36, 37). HS, CS, and DS each have 4, 2, and 3 sulfation locations (38). As a water-soluble polymer, CS may cross-link with other polymers, such as CS, HA, and gelatin, reducing its solubility in water (35, 39–41). This may explain the opposite effect of CS-C when combined with CS-A or/and CS-B (DS) in competition experiments.

**FIG 9:**
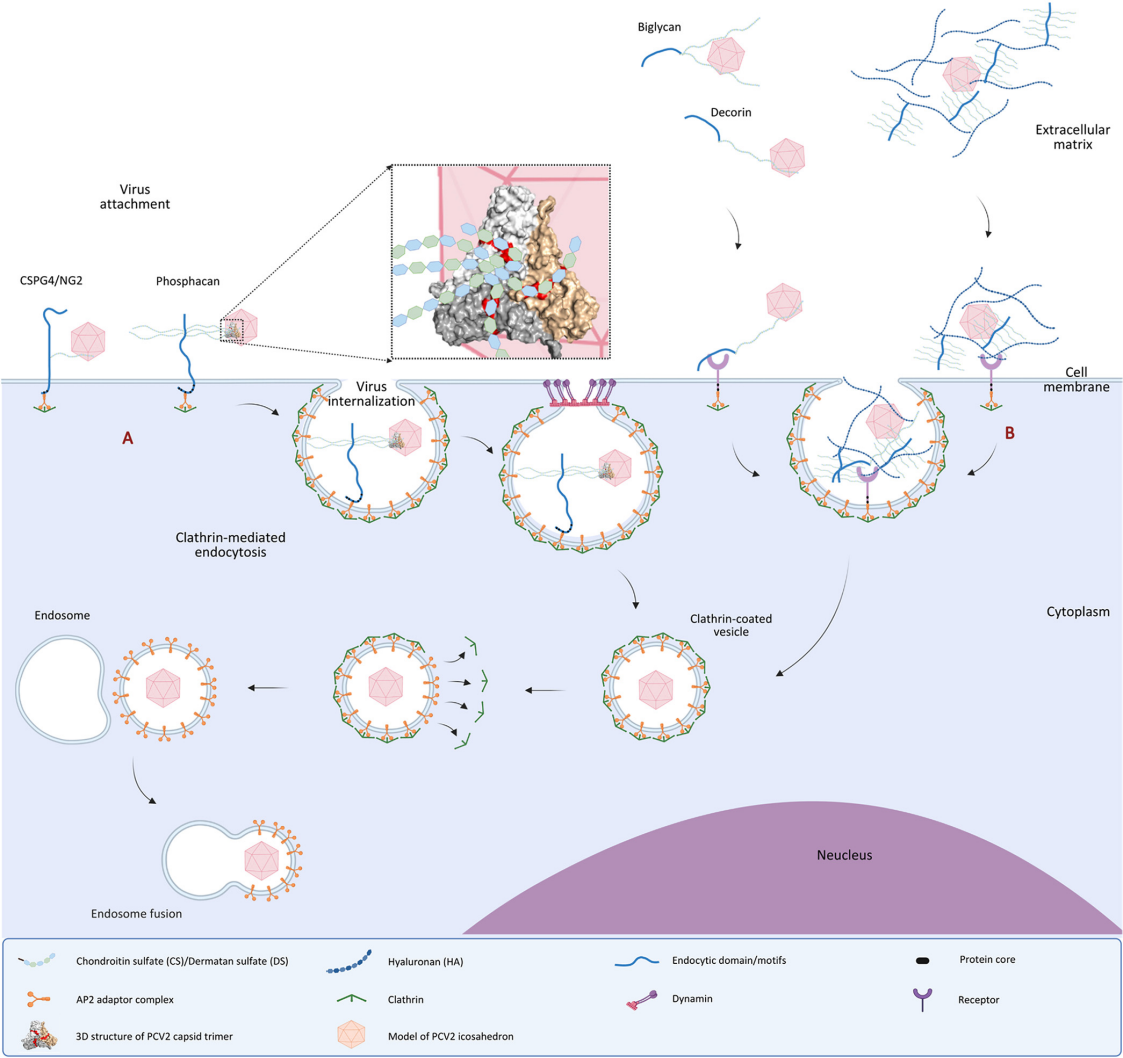
Hypothetical model of PCV2 uptake mediated by phosphacan and other potential molecules. (A) To begin the process of virus uptake, the PCV2 virion electrostatically attaches to the GAG chains of phosphacan, a cell surface proteoglycan. Any 3 chains on phosphacan may interact with the three-wings-windmill-shaped positively charged bands (colored in red in capsid trimer) on the surface of the PCV2 capsids, allowing viral particles to accumulate on the monocyte surface. Given that it has fewer GAG chains and endocytic motifs, CSPG2/NG2 will have poorer binding and internalization efficiency. Extracellular decorin and biglycan may support viral enrichment on the cell surface, and viral delivery to other binding and internalization mediators. Afterwards, the interaction of the viral particle with phosphacan activates the signaling pathway of clathrin-mediated endocytosis, and induces the binding of an adaptor protein complex 2 (AP2) to the phosphacan cytoplasmic tail. APs bind to clathrin, and allow clathrin to involve in forming clathrin-coated vesicles (CCVs) under the facilitation of dynamin. The vesicle then delivers its viral content to endosomes. Then, the endosome with the virus undergoes acidification, followed by transport to lysosomes for further degradation or recycling its content to the cell membrane. (B) Another potential for viral uptake is that viral particles bind with extracellular hyaluronic acids (HA) or CS/DS proteoglycans, triggering internalization by activating the signal of the HA receptor for endocytosis (HARE). Created with Biorender.com.

Blood monocytes have a strong ability to take up PCV2 virions, and slowly disintegrate them (18). During this process, the viral genome becomes released in the cytoplasm. The uptake ability differs among pig breeds with monocytes of purebred Piétrain pigs being more potent than monocytes of Landrace and Large White pigs (18). In this study, antigens remained in the cytoplasm, following PCV2 capsid uptake by monocytes. The internalization and trafficking of the membrane receptors and proteins from the cell surface to the subcellular compartments are mediated by specific short linear amino acid sequence motifs. They are normally located in the cytoplasmic domain of those receptors. YxxØ (where Ø is a bulky hydrophobic residue), [DE]xxxL[LI], and [FY]xNPx[YF] are commonly regarded as the most important clathrin-dependent internalization motifs (42, 43). Cell surface GAGs help viral endocytosis. The GAG chains are usually connected to a protein core with its C-terminus inserted into the cell membrane. On the membrane of the cell surface, CS chains are mainly linked to the protein cores of CSPG4/NG2 (XP_003128533.4), betaglycan (NP_999437), and phosphacan (XP_020947646.1). The intracellular end of the former 2 is a PDZ binding domain, and it is a protein tyrosine phosphatase (PTP) domain for phosphacan (44). The amino acid sequence of their cytoplasmic domain was analyzed and presented in the note of Supplementary Materials. Six endocytic motifs were found in the cytoplasmic domain of phosphacan, while only 2 were found in CSPG4/NG2, and none in betaglycan. In our 3D structural analysis, 3 or 5 of the GAG chains on phosphacan may interact with several special positively charged amino acid bands (three-wings/pentagon) on the surface of the PCV2 Cap trimer or pentamer, and the 3 wings are more functional than the pentagon for virus-GAG interaction. Even though PCV2 only exhibited about 20% colocalization with phosphacan in terms of pixels per colocalized cell, there were still a lot of cells showing colocalization of PCV2 and phosphacan at cell level, with DE222-13 even reaching 90.6%. Indeed, it is still possible that phosphacan is present at low levels that are not visible with the fluorescence microscope. These data support our hypothesis of the role of phosphacan in the PCV2 internalization in monocytes. A hypothetical model for PCV2 binding and uptake mediated by phosphacan (and other potential molecules) is displayed in Fig. 9.

An immunofluorescence assay illustrated the relation between phosphacan and PCV2 uptake. A very high expression of phosphacan was observed in the monocytes from Piétrain pigs; the colocalization between PCV2 and phosphacan was also indicative of the attachment between the GAG chains and the PCV2 capsid, and the internalization mediated by the cytoplasmic tail of the protein core of phosphacan. Lower concentrations of phosphacan in monocytes from Landrace pigs may explain, in part, the lower uptake of PCV2 by monocytes, as mentioned in the publication of Wei et al. (18).

This study illustrated the significance of DS (CS-B) in PCV2 binding and internalization into monocytes. DS (CS-B) is largely distributed in the extracellular matrix. It links to the protein core of some extracellular PGs, such as decorin and biglycan, the 2 best-studied DSPGs (45). Even though they do not mediate viral internalization through endocytic motifs, the special N-terminus location of the GAG binding domain gives the DS chain significant mobility (44). Consequently, DS (CS-B) may help deliver the virions to GAGs linked to transmembrane glycoproteins, such as phosphacan. Further, it has been reported that the hyaluronic acid (HA) receptor for endocytosis (HARE) is the primary scavenger receptor, not only for HA, but also for CS in mammals, leading to the rapid endocytosis of HA and CS via a clathrin-coated pit pathway in an endocytic process (46–49). In this way, PCV2 may also bind to extracellular GAGs and get internalized by HARE.

In summary, our research demonstrated differences in monocyte uptake of different PCV2 strains, and related this with the number and configuration of positively and negatively charged amino acids on the surface of the PCV2 capsid together with certain GAGs on the surface of monocytes. Additionally, phosphacan was hypothesized as an effective candidate to mediate PCV2 uptake by monocytes by analyzing the distribution of positively charged amino acids on PCV2 capsids. The fact that Piétrain pigs have more GAGs and phosphacan on their surface may explain in part the more powerful virus uptake capacity of monocytes compared to Landrace pigs. Since GAGs are only responsible for a part of the binding and internalization of PCV2 in monocytes, other negatively charged molecules and entry pathways should also be considered and studied in future work. Our findings bring a better understanding of the monocytic PCV2 uptake, its role in the pathogenesis of PCVAD, and provide deeper insights into the basis of PCV2 evolution.

## MATERIALS AND METHODS

### Ethics statement.

The protocol for taking blood from pigs was approved by the Ethics Committee on Animal Research and Testing, Faculty of Veterinary Science, Ghent University (EC2013/97).

### Animals.

Nine healthy purebred Piétrain pigs between 7- and 12-weeks old were used as blood donors. All pigs were kept in isolation throughout this study.

### Virus stocks.

Six different PK15-grown PCV2 strains were used in this study. PCV2a-Stoon 1010 was isolated from PMWS-affected piglets in Canada in 1998 (6). PCV2a-1121 was isolated from aborted fetuses in Canada in 2001 (50). PCV2b-1147 originated from a case of PDNS in the UK in 2001 (50). PCV2d1-09V448 was obtained from the lymph nodes of a pig with a high PCV2 viral load (PCVAD^high^) during a PCVAD outbreak in Belgium in 2009 (7). PCV2d2-DE222-13 was isolated from a pig during a PCVAD^high^ outbreak in Germany in 2015 (51). PCV2d2-19V245 originated from the lymph nodes of a pig with a PCVAD^high^ in Belgium in 2019. All viral stocks were filtered through 0.45 μm MF-Millipore membrane filters to remove the cellular debris and large virus aggregates.

### Antibodies.

Mouse monoclonal antibody (MAb) 12E12 (IgG2b) against PCV2 Cap was generated in our laboratory; the antibody titer in the hybridoma supernatant, as determined with an immunoperoxidase monolayer assay (IPMA), was 250 for all strains used in this study (52). Mouse anti-CD172a IgG1 MAb DH59B (Bio-Rad) was used as a cell surface marker of monocytes; mouse anti-HS IgM MAb 10E4 (Amsbio) was used to trace HS PGs. Mouse anti-CS IgM MAb CS-56 (Sigma-Aldrich) reacts specifically with CS-A and CS-C, but not with CS-B (DS). Due to the lack of commercial anti-DS antibodies, rabbit anti-decorin IgG polyclonal antibodies (pAb) (GeneTex) that target the protein core of decorin (the main proteoglycan containing DS), was used in this study to detect the presence of DS on the surface of the monocytes. Phosphacan is also known as protein tyrosine phosphatase receptor type zeta (PTPRZ). Rabbit anti-PTPRZ IgG pAb (Abbexa) was used in this study to detect phosphacan.

All secondary antibodies used in this study were purchased from Invitrogen (Thermo Fisher Scientific), including Alexa Fluor (AF) 488-conjugated goat-anti-mouse IgG2b Ab, AF594 goat-anti-mouse IgG1 Ab, FITC-conjugated goat-anti-mouse IgM Ab, FITC-conjugated goat-anti-mouse IgG Ab, and FITC-conjugated goat-anti-rabbit IgG Ab.

### Glycosaminoglycans and enzymes.

CS-A sodium salt from bovine trachea (Sigma-Aldrich), CS-C sodium salt from shark cartilage (MP Biomedicals), CS-B (DS) sodium salt from porcine intestinal mucosa (Sigma-Aldrich), and HS sodium salt from bovine kidney (Sigma-Aldrich) were included in this study. Heparinase I and III blend from Flavobacterium heparinum (Sigma-Aldrich) and Chondroitinase ABC from Proteus Vulgaris (Sigma-Aldrich) were used for the removal of specific GAG from the cell surface.

### Peripheral blood mononuclear cells isolation and monocytic cell preparation.

Blood was obtained by jugular venipuncture in heparinized syringes. Peripheral blood mononuclear cells (PBMCs) were isolated by density gradient centrifugation on Ficoll-Paque PLUS (Cytiva Life Sciences), according to the procedure of a previous study (18). After 3 washes with cold Dulbecco's phosphate-buffered saline (DPBS), the cells were cultured in a leukocyte culture medium, supplemented with 5% fetal calf serum (FCS, Gibco), 100 U/mL penicillin, 100 μg/mL streptomycin, and 1 μg/mL gentamicin in RPMI 1640 (Gibco). The isolated PBMCs were seeded on glass inserts in wells of a 24-well plate (Thermo Fisher Scientific) at a concentration of 2.5 × 10^6^ cells/mL, and cultured at 37°C with 5% CO_2_ overnight. Non-adherent cells were removed; monocytes remained adherent.

### Quantification of virus particles.

A virus stock was passed through a filter with 0.45 μm pore size, and homogeneously mixed with an equal volume of 0.2 μm red fluorescent Carboxylate-Modified Microspheres (Thermo Fisher Scientific) with a concentration of 10^7^ particles/mL. Mixtures were smeared onto microscopic slides, air-dried, and then fixed for 10 min at room temperature (RT) with 4% paraformaldehyde (PF). Afterwards, mouse 12E12 IgG2b MAb (1:50) was used to stain PCV2 viral particles at 37°C for 1 h, followed by staining with Alexa Fluor 488-conjugated goat-anti-mouse IgG2b secondary Ab (1:200) at 37°C for 50 min. Between and after the incubation, the slides were washed with phosphate-buffered saline (PBS) three times. After mounting the slides, a laser scanning confocal microscope (LSCM, Leica) was used to acquire digital images of red fluorophores and green PCV2 virus particles. Fifteen fields were randomly selected to count and calculate the number of stained PCV2 particles, which is in proportion to the number of fluorophores.

### Uptake of PCV2 particles into monocytes.

Monocytes were incubated with 1.0 × 10^9^ virus particles in a volume of 300 μL on glass inserts in wells of a 24-well plate at 37°C for 1 h. After 3 gentle washes, fresh medium was added to each well for further culture. Cell samples were collected at different time points: 0 min, 15 min, 30 min, 1 h, 2 h, 3 h, 6 h, 12 h, 24 h, 48 h, and 72 h. Cells were fixed with 4% PF, and subsequently permeabilized with 0.1% TritonX-100 for 10 min at RT. To quantify the number of capsids that were taken up by monocytes, immunofluorescent staining were performed. PCV2 antigens were detected by using the specific PCV2 Cap-binding MAb 12E12 (1:50), while monocytes were stained with mouse anti-CD172a MAb DH59B (1:700), in combination with AF488 conjugated goat-anti-mouse IgG2b (1:200) and AF594 conjugated goat-anti-mouse IgG1 Ab (1:300). Hoechst 33342 (1:50) was used for the counterstain of cell nuclei. In between and after the incubation, the inserts were washed three times with PBS. After mounting, 10 random images were taken by LSCM to count and calculate the percentage of PCV2 positive cells, and the number of fluorescing pixels of PCV2 signals by software ImageJ and MATLAB.

### Expression of HS, CS, and decorin (DS) on monocytes.

Before analyzing whether the uptake of PCV2 particles by monocytes is mediated by the binding with GAG chains, the expression of the most common GAGs (HS, CS-A/C, and DS [linked to the protein core of decorin PG]) were determined by immunofluorescent staining. Monocytes were seeded on 24-well plates as described before, and fixed with 4% PF for 10 min at RT. Afterwards, HS, CS, and decorin (DS) were detected by staining with mouse anti-HS MAb 10E4, mouse anti-CS MAb CS-56, and rabbit anti-decorin pAb, respectively. PK-15 cells, which have already been proven to express HS and CS, were stained in parallel as positive controls (24). Cells incubated with only the secondary Ab were used as the negative control. Cell nuclei were co-stained with Hoechst 33342. Images were taken with the LSCM, followed by counting and calculating the number and percentage of GAG positive cells, and the fluorescent area (pixels) per GAG positive cell from 10 randomly selected fields.

### Colocalization between PCV2 particles and HS, CS-A/C, or decorin (DS).

PCV2 binding on GAG-expressing monocytes was evaluated by a co-localization assay of viral particles and GAGs. Using the same methodology as described above, monocytes were incubated with PCV2 strain 1121 or DE222-13 at 4°C for 1 h. Afterwards, cells were fixed, and a double immunofluorescence staining was conducted. The attached PCV2 virions were stained with mouse anti-Cap MAb 12E12; HS, CS, and decorin were stained with specific antibodies, as described above. The percentage of cells showing colocalization was calculated. The fluorescing area (quantified in digital pixels) of individual PCV2 attachment, GAG expression, and the colocalization between the 2 molecules were also quantified by using the colocalization plugins of ImageJ software.

### Evaluation of the role of GAGs in PCV2 uptake of monocytes.

To determine whether GAGs are involved in PCV2 binding and endocytosis, two PCV2 strains (1121 and DE222-13) were selected, and soluble GAGs were utilized as receptor analogues for PCV2 attachment and internalization in this competition experiment, which was modified, based on previous research (24). Firstly, monocytes were seeded on 24-well plates. Next, GAGs were separately dissolved in RPMI 1640 medium, and were mixed in specific combinations. Then, 10^9^ PCV2 particles were mixed with either single or combined GAG(s) at a concentration of 500 ng/mL at 37°C for 90 min. Subsequently, virus-GAG(s) mixtures were transferred to monocytes, and further incubated at 4°C or 37°C for 1 h. Afterwards, the mixtures were removed, and cells were fixed with 4% PF after 3 washes. For internalization experiments, 0.1% TritonX-100 was used to permeabilize the cell membrane for 10 min at RT after fixation. A double immunofluorescent staining was conducted as described before. Briefly, PCV2 particles and cells were stained with Cap-specific MAb 12E12 and CD172a monocyte-specific MAb DH59B, respectively, followed by the conjugated corresponding secondary Ab. Nuclei were co-stained with Hoechst 33342. In between the incubation steps, the cells were washed three times in PBS. The percentage of cells with attached and internalized virus particles was calculated from 10 randomly selected images that were taken by LSCM. The fluorescing area of virus particles per positive cell was counted with MATLAB.

### Effect of GAG removal on PCV2 attachment and internalization on/into monocytes.

Heparinase I & III (Hep) cleaves glycosidic linkages in heparin and HS (53), and Chondroitinase ABC (ChABC) cleaves glycosidic linkages in CS-A, CS-B (DS), and CS-C (54). To remove the GAG chains on the cell surface, monocytes on glass inserts in 24-well plates were incubated with either Hep (2 U/mL) or ChABC (2 U/mL), and diluted in RPMI 1640 medium for 1 h at 37°C, based on previous studies (24, 25), and the concentration of enzymes used in this study did not cause cell death or detachment of cells from the culture plates. After removing the enzymes, cells were washed, and then incubated with PCV2 1121 or DE222-13 at a total number of 10^9^ particles at 37°C or 4°C for 1 h. Hereafter, the viral inoculum was washed off, and cells were fixed with 4% PF. In internalization experiments, cells were permeabilized with 0.1% TritonX-100 after fixation. An immunofluorescence staining was conducted to detect PCV2 particles and CD172a, as described above. The percentage of PCV2-positive cells with or without enzymatic removal was examined, respectively. The fluorescence area of Caps per cell was quantified in pixels.

### Multiple sequence alignment, 3D mapping, and structural analysis of PCV2 capsid proteins.

The Cap sequences from six PCV2 strains used in this study were downloaded from GenBank: PCV2a-Stoon 1010 (GenBank accession No. AAC35310.1), PCV2a-1121 (AJ293868.1), PCV2b-1147 (AJ293869.1), PCV2d-1-09V448 (QCQ78431.1), PCV2d-2-DE222-13 (AJP07089.1), and PCV2d-2-19V245 (USV27802.1). All sequences were aligned by using the ClustalW manner of the MegAlign module of DNASTAR software. Visualization of MSA was conducted by MSAViewer (55).

The 3D structures of Cap from the 6 strains were predicted through the Iterative Threading ASSEmbly Refinement (I-TASSER) server, and the trimeric and pentameric subunits were generated by the SYMMDOCK server. PyMOL was used to display and analyze all the structures. Positively and negatively charged amino acids exposed on the outer capsid surface were defined and quantified.

SASA is the surface area of the biomolecules accessible by the solvent. It is typically calculated by simulating a solvent sphere (such as a water molecule) with a specific radius to ‘probe’ the molecular surface in a ‘rolling ball’ algorithm (56–58). Here, the SASA of positive (SASA^+^) and negative (SASA^−^) residues on the capsid surface were calculated with PyMOL, respectively. The delta of SASA (ΔSASA) was calculated as follows:

$$\Delta \text{SASA }\left({Å}^{\text{2}}\right)\;={\text{ SASA}}^{+}\;-{\text{ SASA}}^{-}$$

### Expression of phosphacan on monocytes.

Phosphacan is a rich-GAG PG that normally possesses 2 to 5 CS chains (59, 60), which may bind to PCV2. Before analyzing whether phosphacan mediated PCV2 binding to monocytes, immunofluorescence staining was performed to examine the expression of phosphacan on the cell membrane of blood monocytes. As described before, monocytes were seeded on 24-well plates and fixed with 4% PF. HepG2 cells were used as a positive control. Rabbit anti-PTPRZ IgG pAb was used to detect phosphacan expression, followed by the conjugated corresponding secondary Ab. Cell nuclei were co-stained with Hoechst 33342. In between the incubation steps, the cells were washed three times in PBS. The percentage of cells showing phosphacan expression, and the fluorescent pixels per phosphacan positive cell were quantified from 10 randomly selected images that were taken with the LSCM.

### Immunofluorescence staining and colocalization analysis of CS and phosphacan.

Phosphacan has a core protein linked with 2 to 5 CS chains. To verify the link between CS and phosphacan, a double immunofluorescence staining was performed. Fixed monocytes were incubated with mouse anti-CS-IgM MAb and rabbit anti-PTPRZ-IgG pAb, followed by incubation with FITC-conjugated goat-anti-mouse IgM and AF594 conjugated goat-anti-rabbit IgG. Nuclei were co-stained with Hoechst 33342. In between the incubation steps, the cells were washed three times in PBS. The immunofluorescence signals of CS and phosphacan were detected, and 20 randomly selected images were taken by LSCM. The percentage of cells showing CS- phosphacan colocalization was calculated.

### Colocalization analysis of PCV2 and phosphacan on PCV2 binding to monocytes.

To demonstrate whether PCV2 particles attach to monocytes via binding to phosphacan or not, immunofluorescence staining was performed to analyze their colocalization. PCV2 particles and phosphacan were stained with mouse Cap-specific MAb 12E12 and rabbit anti-PTPRZ IgG pAb, respectively, followed by the conjugated corresponding secondary Ab. Nuclei were stained with Hoechst 33342. In between the incubation steps, the cells were washed three times in PBS. The LSCM was used to detect the fluorescence signals. The percentage of cells showing PCV2 binding, phosphacan expression, and their colocalization were quantified, respectively, in 20 randomly selected images.

### Statistical analysis.

Counting cells and calculation of fluorescent density (pixels) were performed with MATLAB (MATrix LABoratory). Colocalization of immunofluorescence assay was analyzed by ImageJ software (National Institutes of Health). Data were analyzed using GraphPad Prism 9.0 software (LaJolla). *, *P < *0.05 was considered statistically significant.

### Data availability.

Original data supporting the findings of this study are included in the paper and supplemental materials. Further inquiries can be directed to the corresponding author without reservation.
