# Supplementary material for: PCV2 Uptake by Porcine Monocytes Is Strain-Dependent and Is Associated with Amino Acid Characteristics on the Capsid Surface
Source: Microbiol Spectr. 2023 Jan 31;11(2):e03805-22. doi: 10.1128/spectrum.03805-22 (PMC10100887; doi:10.1128/spectrum.03805-22)
Supplement: Supplemental file 1 — Supplemental note, Fig. S1 and S2, and Table S1. Download spectrum.03805-22-s0001.pdf, PDF file, 0.4 MB [file spectrum.03805-22-s0001.pdf]

Supplementary Materials

● Supplementary Note

Endocytic signal domains/motifs in three proteoglycans that are carrying chondroitin sulfate (CS) side chains

Three motifs have been shown to be both necessary and sufficient for the uptake of cargo into clathrin-coated vesicles (CCVs): **YXXΦ** (where Φ is a bulky hydrophobic residue), **[D/E]XXXL[L/I]** and **[F/Y]XNPX[Y/F]**. Their presence in the cytoplasmic tail of phosphacan, chondroitin sulfate proteoglycan 4 and betaglycan have been analyzed.

(1) Phosphacan/receptor-type tyrosine-protein phosphatase beta isoform X1 [Sus scrofa]

NCBI Reference Sequence: XP_020947646.1

1 meagfylavl tcflvgnseg fqvvhvqkqq clfrnkrvvv glcngtslnq qwmwtkngkl
61 lhvksalclg vsdssggpsq saifapcsqa prwtchekeg flevenaslf lkkqgfkvvv
121 kkgrkylhsw mkidvneegk pvieslcsek aglgaevsvr strdttppqi pttfnalpys
181 pghlirntte aftrsateny sqnssqrqhp slqtagitsw vpwttqpfss tteetglgeq
241 vrcnftltes rvsnrsvslq wrtlaspcnf sltyssdtsr vawchpvrid nttygcnpkd
301 lqagtiynfr ivsltgeekt vvlqtdplpp arfevskekt tstslhvwwt pssgkvtwye
361 vqllddnqkt qgaqiqerta rneytflnlt agnkyniait aisgdkrsft iytngstvps
421 pvkdvgisae tnslliswsr gpgnleryhl mlmdkgilvh sntvdkstts yafhgltpgh
481 lynvtivtea aglqnyrwkp arttpmevsn lkvtndgtmt slkakwqrps gnvdfynltl
541 shqgtvkesr ilaphitetq fknltpgrly qvtiscvsge lsahktavgr tvpekvgnle
601 ansngsmrsl vvswsppagd weqyrillln dslvllnitv gkeethyvid evelipgrqy
661 evevtvesgn lknskrchgr tvpmavlqlr vkhanetsls imwqapaaew ekyiislddr
721 dlliihkslp rdakeftftd lvpgrkykaa vtsisgdlkn ssstkgrtvp aqvtglrvan
781 qgttsslftn wthapgdref yqvllihenv viknesvsse tsrysfhslk sgslysvvvt
841 tvsggissrq vvvegrtvps svsgvtvnns grndylsisw lpapgdvdny vvtlshdsrv
901 vqslviaksv secsfssltp grlynvtitt rsgkyenhsf gqertvpdkv qgvsisnsar
961 sdylkvswvh atgdfdhyev tiknknnfiq tktipksene cvfvklvpgr lysvtvstks
1021 gqyeaseqgn grtipeavkd ltlrnrsted lqvtwtradg dvdqyeiqll fndmkvfpsf
1081 hlvntateyr ftsltpgrqy kilvltvsgd vqqsafiegf tvpsvvknir vspngatdsl
1141 tvnwtpgggd vdsytvsafr qsqtvesqti pkhvsehtfh rleageqyqi liasvsgslk
1201 nqievfgrtv pasvqgimad naysshsliv swqkavgvve rydilllsen gillsnisea
1261 attkqhkfed lipgkkykiq iltvsgglfs keaqtegrtv paavtnlrit enstghlsfs
1321 wtpskgefkg yniflynpdg tlqeraqidp tvqsfsfqnl lqgrmykmvi vtcseelsne
1381 slmfgrtvpa svsnlkgsnr nmtdslwfsw spasgdfdfy elilynpngt kkenwkdknl
1441 tewrfhglip grkytlcvvt hsgdlsnkvl gesrtapspp slmsfadvan tslaitwkgp
1501 pdwtdyddfe lqwfprdait vfnpynnrks egrivyglrp grsyqfsvkt vsgdswktys
1561 kpvfgsvrtk pdkiqnlhcr pqnstaiscs wippdsdfdg ysiecrkmdt qevefsrkle
1621 kekslInimm lvphkrylvs ikvqsagmts evvedstitm idrpppppph irvnkkdvli
1681 skssinftfn cswfsdtnga vkyftvvvre adgsdelkpe qqhplpsyle yrhnasirvy
1741 qtdyfaskca espdsnsksf niklgaemes lggkcdpnqq kfcdgplkpr tayrisiraf

1801 tqlfdenlke ftkplysdtf fslpittese plfgviegvs aglfligmlv avvalficrq
1861 kvnhgrerps arlsirrdrp lsvhlnlgqk gnrktscpik vnqfeghfmk lqadsnylls
1921 keyedlkdvg rnqpcdiall penrgknryn nilpydasrv klsnvdddpc sdyinasyip
1981 gnnfrreyia tqgplpgtkd dfwkmaweqn vhnivmvtqc vekgrvkcdh ywpadqdsly
2041 ygdlilqmls esvlpewtir efricseeql dahrlirhfh ytvwpdhgvp ettqsliqfv
2101 rtvrdyinrt pgagptvvhc sagvgrtgtf ialdrilqql dskdsvdiyg avhdlrlhrv
2161 hmvqtecqyv ylhqcvrdvl rarklrseqe nplfpiyenv npeyhrdagy srh

(2) Chondroitin sulfate proteoglycan 4 [Sus scrofa]

NCBI Reference Sequence: XP_003128533.4

1 mrsgprlprp apalalaltf avlarpaata sffgenhlev platalpdid lqlefstsqp
61 eallllaagp vdhlllqlys grlqvrlilg reevrlqtla emllsdsvph tvgltvsdsw
121 aslsvdglln asalvqggpl evpyglflgg tgsldlpylr gasrplrgcl htatlngrsl
181 lrpltpdvpe gcaeefsagd dvalgfsgph slaafpawgt qdegileftl ttrsrqapla
241 fqaggrhgdf iyvdifeghl ravvekgqgt vllhnsvpva dgqphevsih vdahrleisv
301 dqyptrtsnr gvlssleprg slllggldae asrplqehrl glavnvsllg cledlsvngq
361 rwglrdallt rsmaagcrle edeyeedayg pyealstlap eawpamelpe pcvpepglpp
421 ifanftqllt tsplvvaegg tawlewrhvq ptldlseael rksqvlfsvs rgarhgelel
481 dipgaqarkm ftlldvvnrk aryvhdgsed tsdqlvlevs vttrgpvpsc lrrgqtyilp
541 iqinpvndpp rvifphgslm vilentqkpl gpeifqaydp dsacegltfq llgapaglpv
601 errdqpgepa tefscrelea gslvyvhrgg paqdltfrvs dglqasppat lkvvavrpai
661 qvrhntglrl aqgsaapvsp anlsvetnav gqdvsvlfrv tsplqfgelq kqgaggaega
721 ewrpiqafhq rdveqgrvry lstdpqhrte dtvesvalev qvgpetlsnl sfpvtvqrat
781 vwllrlepld tqntrqkalt tahleatlee agpnpttfhy evvqapkkgn lrlqgtrlsd
841 gqgftqddlq agrvtygata raseavedtf rfrvtapphf splytfpihi sgdpdapvlt
901 nvllsvpegg egvlsadhlf vkslnsasyl yevmerprhg rlawrgaedk vttvtsftne
961 dllhsrlvyq hdgsettedd ipfvatrqge gsggmaweev rgvfrvaiqp vndhapvqti
1021 srvfhvargg rrllttddva fsdadsgfad tqlvltrkdl lfgsivavde ptrpiyrftq
1081 edlrkrrvlf vhsgadrgwi qlqvsdgqhq atallevqas epylrvtngs slvvpqggqg
1141 tidtavlhld tnldirsgde vhyrvtagph wgqllragqp atsfsqqdll dgailyshng
1201 slsprdtltf sveagpvhtd atlqvtiale gplaplqlvq hkkiyvfqge aaeirrdqle
1261 aaqeavpptd imfsvrtpps agylvmlshg ttaaeppsld pvhsfsqeav dagrvlylhs
1321 hpeawsdafs ldvssglgap lqgirmelev lpaaipleaq nfsvpeggtr tlappllrit
1381 gpyfptlpgl dlqvlepprh galrreegpp dgalstfswr eveqqlihyv hdgsetlads
1441 filvanasem drqshpvaft itilpvndqp pvlttntglq mwegatvpip tealrgadnd
1501 sgpedlvytl eqpingrvll rtepgtevhs ftqaqldggl vlfshkgald ggfrfslsdg
1561 ehtspghffr vvaqkqplls legsrtltvc pgsvqplssq slrasssagt dprhllyqvv
1621 qgpqlgrlfh tqrgpageal vnftqaevya gnvlyehemp pepfweardt lelqlssppa
1681 pdvattlavs vsfeaacpqr psrlwrnkgl wvsegqraei ttaaldasnl lasvpspqrl
1741 ehdvlfqitq fptrgqllvs eeplhagrph flqselasgq lvyahggggt qqdgfrfrah
1801 lqgpagtlva gphtseafai tvrdvnerpp qpqasiplrl trgsrsplsr aqlsvvdpds
1861 apgeieyevq raphngylsl pgaspgpvtr ftqadvdagr lafvangssv agifqlsvsd
1921 gaspplpmsl vvdvlpsaie vqlraplevp qavgrsslsr qqlqvvsdre eldaayrltq

1981 gpryghllvg gqpatafsql qvdqgevvfa ftnfssphdq fsilalarga nasatvnvtv
2041 rallhvwtgg pwpqgatlrl dptvldagel anhtgsvprf rllagprhgr vvrvprarte
2101 prggqlveqf tqqdledgrl glevgrpegs ppgpagdslt lelwargvpp avasldfate
2161 pynaarpysv allslpeaar teagkpesst ptgkpgpaas spvpavargg flgfleanmf
2221 siiipvclvl lllalilpll fylrkrnktg khnvqvltak prnglagdne tfrkvdpgqa
2281 ipltavhgqg ppqggqpdpe llqfcrtsnp alkngqywv

(3) Betaglycan/transforming growth factor beta receptor type 3 precursor (TGFβ type III receptor) [Sus scrofa]

NCBI Reference Sequence: NP_999437.

1 mtlhcvvalf alissclata gpepgvqcal spvnashpvq almesftvls gcasrgtmgr
61 pqevhvlnlr aadqgpgqrq sevtlhlnpi ssvhihhkpv vfllnspqpl vwhlkterla
121 vgvsrlflvs egsvvhfssg nfslsaetee rnfphgnehl lnwarkeyga vtsftelkia
181 rniyikvged qvfpptcsig knflslnyla eylqpkpaeg cvvsgrpqek evhiieliap
241 nsnpysafqv diiidirpsr kdpelvknli lilkckksvn wviksfdvkg nlkvlapnsi
301 gfgresersm imtksvrddi pstqekllrw aldngyspvt sytvapvanr fhlrlennee
361 mrdeevhtip pelqilldpg alpvldhpps gegaarhggl pfpfpyiprr grqdggkdrl
421 prpkdpvvps iqllpgprep qeaqgsrdva lsvrcdsekm lvavekdsfq asgypglelt
481 lldptckakt ngthfilesp ldgcgtrhrr sapdgvvyyn siviqappsg dssgwpdgye
541 dlesgdngfp gdvdegdval ssrpelvvfn cslrparhps raqdpptrnv tfsmdlyttd
601 lflapaqgvf svaenghvyv evsvtkadqe lgfaiqtcfi spysnpdrms dytiienicp
661 kdesvkfydp krvhfpipqa etdkkrfsfv fkpvfntsll flqceltlct krekepqklp
721 kcvlpdeact sldasmiwam mqnkktftkp lavihhevqf kgpstkesnp isppifhgld
781 tltvmgiafa afvigalltg alwyiyshtg dsagrqpvpt sppasenssa ahslgstqst
841 pcssssaa

*The intracellular domains are highlighted in grey, and the endocytic motifs are printed in red.

- **Supplementary Figures**

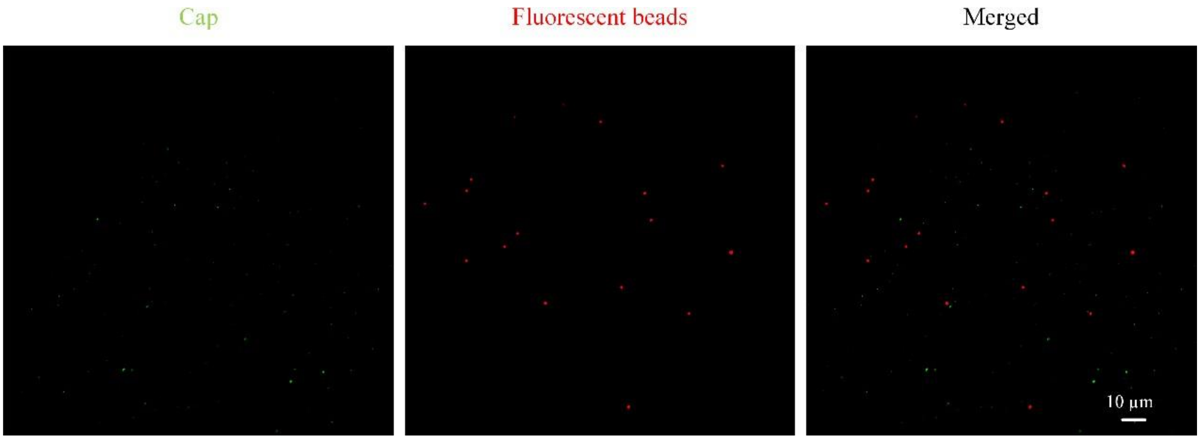

Fig. s1. Representative fluorescent confocal images of immunostained PCV2 particles in green and 200 nm red-fluorescent carboxylate-modified microspheres acquired at the same magnification.

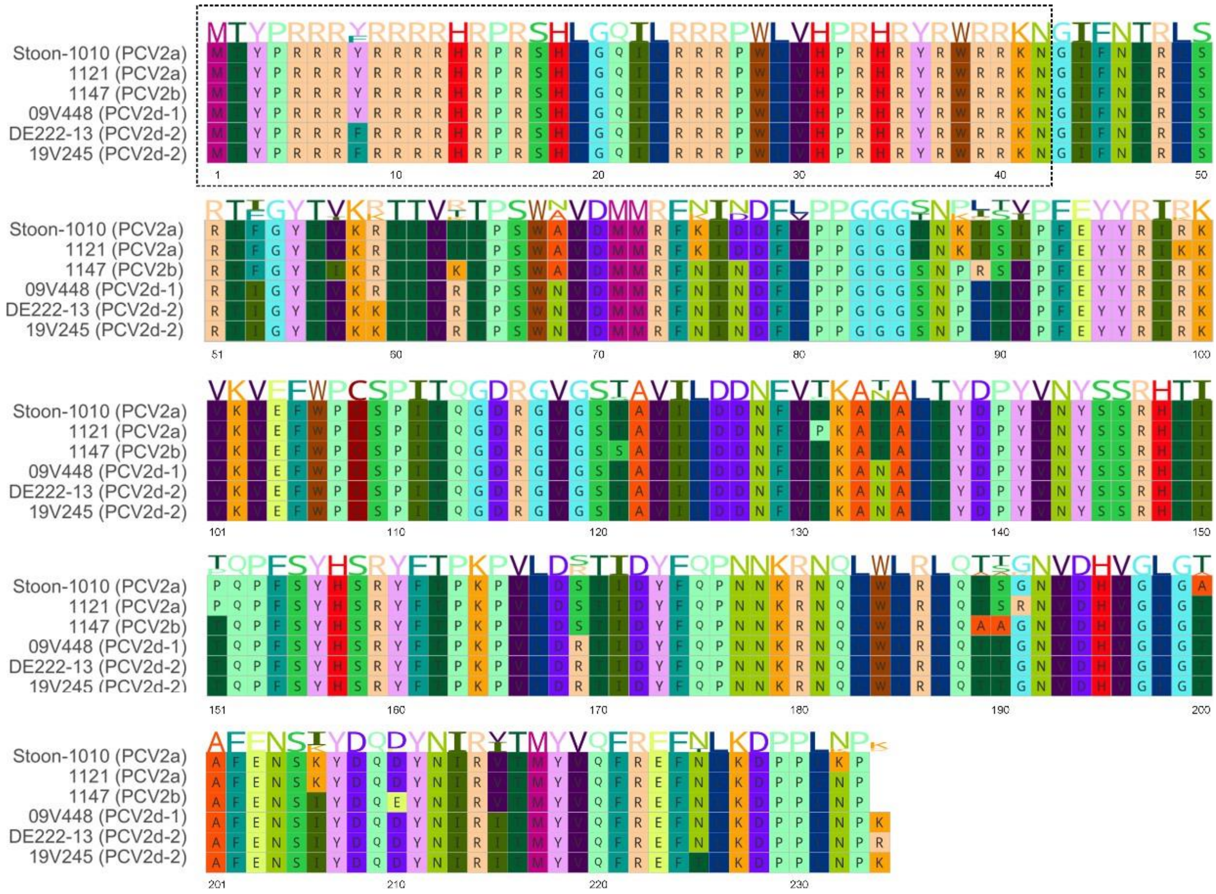

Fig. s2. Amino acid sequence alignment of the capsid proteins of the PCV2 strains Stoon-1010 (PCV2a), 1121 (PCV2a), 1147 (PCV2b), 09V448 (PCV2d-1), DE222-13 (PCV2d-2), and 19V245 (PCV2d-2). Conserved and variable residues in the capsid proteins of six PCV2 strains were depicted in this chart which was generated using MSA Viewer. Flexible N-terminal ends of the capsid proteins (1-42 aa) were framed by a black dashed rectangle.

Supplementary Table

Table s1. Reduction of PCV2 attachment and internalization by GAG competition and enzymatic GAG removal

Treatment	Attachment		Internalization	
GAG competition	1121 (PCV2a)	DE222-13 (PCV2d-2)	1121 (PCV2a)	DE222-13 (PCV2d-2)
CS-A	35.2 ± 15.7	45.0 ± 27.4	25.9 ± 12.0	23.7 ± 2.6
CS-B (DS)	35.0 ± 43.4	21.4 ± 46.1	69.9 ± 17.2	54.4 ± 22.3
CS-C	25.9 ± 27.5	31.3 ± 10.8	9.8 ± 39.0	22.9 ± 19.5
CS-A + CS-B (DS)	82.4 ± 4.2	88.6 ± 5.6	30.4 ± 11.6	57.6 ± 20.7
CS-A + CS-C	64.5 ± 10.7	63.6 ± 5.2	20.0 ± 39.8	9.7 ± 33.0
CS-B (DS) + CS-C	57.8 ± 21.2	52.4 ± 5.6	19.7 ± 46.9	32.3 ± 28.9
CS-A + CS-B (DS) + CS-C	76.2 ± 19.8	59.6 ± 21.5	45.8 ± 23.8	75.7 ± 4.1
HS	-1.0 ± 10.3	13.0 ± 11.1	-13.0 ± 61.0	12.5 ± 53.6
GAG removal				
ChABC	41.9 ± 39.7	61.9 ± 19.0	40.5 ± 39.2	84.0 ± 6.4
Hep	-8.2 ± 82.4	0.7 ± 37.7	-4.3 ± 59.3	13.5 ± 18.5
ChABC + Hep	45.5 ± 68.8	52.4 ± 19.6	57.4 ± 32.1	76.2 ± 10.9

*Data: Mean ± SD
